# Mr Carmichael's Essay on Venereal Diseases, and the Uses and Abuses of Mercury in Their Treatment

**Published:** 1825-10-01

**Authors:** 


*An Essay on Venereal Diseases, and the Uses and Abuses of Mercury in their Treatment; illustrated by Engravings of the different Forms of Venereal Eruptions.* By RICHARD CARMICHAEL, M. R. I. A. &c. &c. Second Edition. London, 1825, pp. xvi. 376.

LET it not be imputed to defect in the constitution of our nature, that this same vile syphilis, the squalid offspring of sensuality and pollution, should have called forth a greater amount of intellectual emulation, and been oftener made the subject of philosophical and experimental research, than many other baneful maladies that prey, as if by preference, on the vitals of the amiable and the innocent. It is, in sooth, an enticing, though an unholy theme; but we care not, at present, to retrace the oozy labyrinths of its medico-chirurgical history.* On this occasion, therefore, we shall not stay to attempt proving that syphilis, and the actions in which it originates, were rife before the flood, and constituted a principal cause of that overwhelming catastrophe:—nor deem we it expedient to discuss the probability of Moses, both as a legislator and physician, having been acquainted with its nature, and its cure:—as little shall we dissent from the creed of those who believe its existence to have been more ancient than the return of Columbus, in January 1493, from the discovery of the West Indies, and his first expedition; or hold as doubtful the doctrine, that John Arden's "brenning of the pintle," and the "infirmitas mulierum nefanda" in his Grace the Bishop of Winchester's "Customarie Book," were the true lues, carrying with it a secret taint:—neither shall we make it questionable whether Joseph Grunbeck, 1496, gave the first systematic account of this sordid malady, on the Continent; or Francisco de Villabolo's, 1498, the first in Spain; and Jacque de Bethencourt, 1527, the first in France; or William Clowes, 1575, the first in England; and the facetious Peter Lowe, (the father of the faculty of physicians and surgeons in Glasgow) the first, 1596, in Scotland:—no more do we desire to disturb the astrological chronology of Wendelin Hock, when he states, 1502,

* The works of Luisinus, Astruc, Girtanner, Gruner, Ploucquet, and Reuss, will greatly facilitate the views of those in the profession who may be desirous of investigating the history, pathology, and modes of treating the syphilitic diseases.

that the "Mentagra," *must* have primarily germinated in Europe, in 1483, become frequent in 1484, and grown very general in 1487, because the "new disease" *must* have been produced by the junction of Jupiter, Mars, Mercury, and the Sun, in the sign Libra, which, in his mind, is the "very dwelling-place of diseases;" or to dissent from that of others, who fix on 1493 or 1494, as the epoch when the "reign of syphilis" was completely and extensively established:—neither shall we undertake to shew that Juan Almenar, 1502, was the first to conceive the idea of distinguishing the venereal cases in which mercury is useful, from those wherein its effects are pernicious:—nor shall we indulge a disposition to controvert the theory of the excellent and venerable Leonicens, when, 1497, he assumes as certain, that syphilitic affections were originally determined by insalubrious vegetable emanations, consecutive to the overflowings of the Tiber, the Po, and other Italian streams:—nor shall we dispute the reasonableness of Jacque Jonvenncel's conclusion, wherein he declares, 1572, that syphilis is absolutely a "cluster of diseases:" or question the philosophy of Jean Riolan, though he affirm, 1574, that there is "something divine in the pestilent lues:" or wittingly deprive John Bernhard Oelffen of his discovery, 1721, that this malady depends on derangement of the "seminal atoms:"—nor yet would we quarrel with Peter Paumier, 1596, and George Arbaud, 1606, when they find themselves led, by fair logical induction, to deny that mercury is an antidote to venereal disorders; nor with Robert Patius, 1649, and Desiderius Claudius Fremond's, 1727, equally just corollary, that mercury is the *only* antidote to syphilis:—neither shall we essay to demonstrate, by the rules of Aristotle, that Jerome Tracastor's "Syphilis," 1503, is a regular and most beautiful poem on the most loathsome ("*fœda lues*,") and execrable of all diseases; or that the burlesque rhymings of Giovanni Battista Lalli, on the same sublime subject, 1629, abound with mirth, melody, and medical science:—nor is it our intention to gainsay the declamations of David Abercromby, 1684, and Francois Chicoynean, 1718, and Dr. Willoughby, 1723, against the advantages of mercurial ptyalism; or to divest *Doctor* J. Sintelaer,* though a knave and a quack, of the honour of having been, 1709, the first English "antimercurialist:"—nor shall we tarry to investigate the accuracy of Jesse Foote's new, 1790, discovered, fact, that a person is insusceptible of a

* For an account of this important personage, and his proceedings, our readers may consult the Number of this Journal for December, 1821.

secondary infection from syphilis, while he remains uncured of the primary disease; or that of the "communications" which, in the mind of Dr. Beddoes, 1800, demonstrated the efficacy of nitrous acid, in every form of the syphilitic malady:—nor list we to join, at this time, in lauding Sir James M'Grigor, and Dr. Thompson, and Dr. Hennen, and the military surgeons, for their very praise-worthy contributions to the arrangement of a just antisyphilitic practice:—neither is it our object to re-consider the first edition of Mr. Carmichael's "novel views which gave the profession not a little perplexity;"—nor shall we venture to impugn the dogmas of his "Athenian" reviewers, or to suggest the possibility of there having been, perhaps, as much of the "ultrices iræ" as of "philosophy benign" in his "Reply" to these oracular judges:—but we shall proceed, forthwith, to attempt an analytical exposition of what we regard as most valuable in his doctrines and practice; reserving to ourselves, however, the privilege of indulging in occasional remarks, which we desire to have considered as the expression of our particular sentiments.

Mr. Carmichael's object, in this treatise, is declared to be twofold,—to elucidate an important class of diseases that have been confounded with syphilis in previous writings, as well as practice; and to discriminate the cases in which mercury may be advantageously employed, from those where it ought to be avoided, as a far greater evil than the poison against which it is intended to act as an antidote. The discussion of these intricate questions occupies seven chapters, each of which we propose to take successively under consideration.

Chap. I.—*Observations on those Morbid Poisons which stand in nearest Relation to the Syphilitic, and evidence of the Existence of Venereal Diseases which do not arise from that Poison.* This chapter is introduced with the statement of a *physiological fact*, which observation undoubtedly confirms, and of an *induction*, concerning the legitimacy of which, a difference of sentiment may possibly be entertained. We shall transcribe the fact and the induction; and, with one practical remark, pass on to the other topics with which Mr. C's first section is occupied.

"It is a curious fact," our author begins, "that morbid poisons which excite considerable fever, such as the small-pox and measles, yield to the powers of the constitution, and are capable of a spontaneous cure; while, on the contrary, the poison of syphilis, which produces scarcely any fever, or one so low as in general to escape observation, yields so slowly and imperfectly to those powers, as commonly to require, for its extinction, the intervention of art. In the former instance, the increased action of the system is sufficient to overcome the poison; but, in syphilis, it seems to be nearly, if not altogether insufficient. Thence, it would appear, arises the necessity, in this disorder, of artificially raising an action, by means of mercury, which, though capable of superseding the influence of the poison, does not, however, extinguish, like the natural fever in small-pox, the susceptibility of receiving the disorder again."

Now, granting Mr. C's first principle, as we very readily do, we cannot, nevertheless, perceive any thing like a philosophical connexion between it and his consequent doctrine, "the necessity of artificially *raising* an action by means of *mercury*:" in our mind, it neither authorises us to teach, that an action should be *raised*, nor, though such action were required, that it ought to be *raised* by the specific operation of *mercury*. From the fact, then, as a first principle, it is quite evident that we cannot, legitimately, come to other than this conclusion,—that "in this disorder," the morbid should be *changed* only, and healthy action restored: but whether this *change* can be accomplished by the salutary influences of the "sacred wood," or of sarsaparilla, or mercury, or of rest and regimen, can be discovered purely by experience: *i. e.* the mode of practice,* suggested by this view of the first principle, must be entirely empirical. Every one knows, of course, that incessant changes naturally take place in the animal economy, through the processes of assimilation, absorption, and excretion; and that the end of these changes is, the appropriation of such organizable elements as may be subjected to the action of parts, for the purpose of being made conducive to their sustenance and vitality, and, at the same time, the rejection of whatever, in these parts and the system, is preternatural, unsalutary, or has become useless; that living nature, in short, unceasingly labours to maintain the healthy state of existence, and to resist or expel disease. Such being the case, the syphilitic poison, however introduced, must have the effect of exciting in the system a self-defensive re-action, so to speak, having for its chief object, the decomposition or expulsion of the poison, and the consequent re-establishment of health; and it is manifest, that the means of attaining this end shall undergo many modifications from the circumstances, whether incidental or constitutional, of those individuals to whom the poison has been com-

* More than four years since, we expressed sentiments regarding this question which we have not yet found reason to qualify or renounce. They stand in the Number of this Journal for March, MDCCCXXI, p. 569.

municated. By adopting this view of the question as a practical principle, then, we shall be better able to reconcile the discordant results of the same remedies in the same form of disease, and to explain the reason of the same disease being readily cured by various and even very dissimilar methods and means of treatment.

Proceeding now with Mr. C. to a consideration of those morbid poisons which stand in nearest relation to the syphilitic, we find him making the yaws, sivvens, the Canadian disease, the northern leprosy, and the button scurvy of Ireland, the subject of inquiry. His account of each of these singular affections is neat and perspicuous; but we cannot enter into a detail of them; his concluding remarks on the yaws, may be taken in illustration of his sentiments regarding the nature and treatment of the rest.

"Upon reading them (Dr. Bateman's observations on yaws,) I was forcibly struck," says Mr. C. "with the strong coincidence which exists between that disease and the constitutional form of some of those complaints which have hitherto been confounded with syphilis. This coincidence is not only striking, with regard to their form, the eruptions being alike composed of papulæ and pustules, and attended with pains in the joints; but also in the circumstance that, like the exanthemata, they will run their course, and not relinquish the constitution till they have completed their various stages. We also find that the exhibition of mercury, in those resembling (syphiloid) diseases, may suspend the disease itself, and clear the skin of the eruption, but will leave the patient still impregnated with the virus, which evinces its presence as soon as the mercurial irritation has subsided, by a re-appearance of the eruption, or affections of the deep-seated parts."

Mr. C's sketches of the "resembling diseases," will be perused with great interest and advantage: we are decidedly of opinion with him, that not one of them has any natural or necessary relation to the syphilitic taint, though any of them in a state of complication with it may be occasionally detected in practice. The remaining portion of the chapter is occupied with testimonies of the existence of syphiloid diseases before the introduction of syphilis. As these testimonies are chiefly taken from Astruc, Becket, Hunter, Adams, Abernethy, and Bateman, we shall content ourselves with referring our readers to the original writings, and finish this branch of the subject with extracting Mr. Carmichael's concluding paragraph.

"Having, as I conceive," p. 40, "adduced sufficient evidence to prove, that ulcers in the generative organs were at all times common before there was, in this part of the world, any acquaintance with syphilis; and that these ulcers were frequently followed by constitutional disorders; we must acknowledge," says he, "the necessity of discriminating them from those of true syphilis, and from each other, and not condemn all, however unlike, to a similar mode of treatment, because they happen to be found on the same parts, and are produced by the same kind of communication. We might, with as much consistency, treat all ulcers of the throat alike, whether arising from scrofula, scarlatina, or simple inflammation; yet, strange to tell, at this improved period of surgery, and notwithstanding the valuable observations of Mr. Hunter, Mr. Abernethy, and Dr. Adams, it is too generally the practice to treat every ulcer on the genitals as syphilitic, whatever may be its appearance, character, or distinction."

CHAP. II. *General Observations on Venereal Diseases: their Specific Distinctions, and Appropriate Mode of Treatment.*—After alluding to the extraordinary mass of evidence collected by the Army Medical Board, on the non-mercurial mode of treatment, Mr. C. condescends to this expression, to which, we suspect, there will still be some objections.

"This much," says he, p. 47, "I believe I may venture to assert, that ample experience has been afforded to prove that every form and stage of venereal disease are capable of being cured without the aid of mercury; and that nodes and affections of the deeper seated parts seldom or never occur in those cases where mercury *has not been employed.*"

To the following statement, however, it is manifest that fewer exceptions, at least rational exceptions, can be taken;

"I have said above," Mr. C. adds, "that the non-mercurial mode of treatment in the cases collectively considered, has been more slow in effecting a cure, than where mercury has been employed; but, if we withold that medicine when its employment may be dispensed with as useless, or avoided as dangerous, and if we have recourse to it where its adoption is likely to be attended with advantage, I shall venture to assert, that the cure in all cases, collectively considered, will not only be far more certain, but by many degrees more rapid, than by following either of the sweeping systems of exhibiting or with-holding mercury in every case."

Having made these previous statements, Mr. C. enters into a discussion, critical, practical, and pathological, from which we may deduce in general,—that he disbelieves the doctrine of mercury being the only remedy for every form of venereal disorder, gonorrhœa excepted; that he regards the term *syphiloidal*, as a plausible though delusive epithet, adapted well enough to conceal professional ignorance, but leading by direct tendency to uncertain or dangerous practice; and that, from the difference that exists in the appearance and progress of certain groups of concomitant symptoms, he presumes the existence of a plurality of venereal poisons. In support of this last conclusion, he adduces the indisputable variety of character exhibited in primary ulcers and venereal eruptions; the latter being so numerous as to afford specimens of almost all the different orders of cutaneous diseases. He also allows considerable weight to the historic evidence detailed by a multiplicity of authors, testifying that infectious venereal diseases existed at all times previous to the introduction of syphilis, towards the close of the 15th century; and thus reasons,—if we were to admit but one venereal poison, we must conclude that the regularity of the progress, and uniformity of the symptoms, which are observed in all other morbid poisons, do not appertain to this, and this alone; thus making an unreasonable and unwarrantable exception to a universal law of nature. After arguing, and we think not unsuccessfully, that the plurality of venereal poisons is confirmed by the variety of primary ulcers, and still more so by the multiplicity of constitutional eruptions, he advances these very judicious remarks, which we have pleasure in transcribing.

"This view" (the distinction between a papular and the pustular terminating in ulcers) "may conduce to satisfy us that the powers of the constitution are much more restricted in modifying the symptoms of a morbid poison than is supposed by those who adhere to the general opinion, that the same virus may produce in one person the scaly lepra; in another, papulæ that terminate in desquamation; in a third, pustules, the precursors of malignant ulcers; and in a fourth, tubercles, exhibiting a diversity which does not occur in the eruptions of small-pox, measles, scarlatina, and other morbid poisons—at the same time, forgetting that all are equally subjected to the influence of the constitution."

"From an attentive consideration of a vast number of cases," he adds, p. 53, "I find strong grounds for concluding, 1st. That the syphilitic chancre is attended by the scaly eruptions, lepra and psoriasis, an excavated ulcer of the tonsils, and pains and nodes of the bones; 2nd. That the simple ulcer, without induration, raised edges or phagedenic surface,—gonorrhœa virulenta, and excoriation of the glans and prepuce, are followed by a papular eruption, which ends in desquamation, pains in the joints resembling those of rheumatism, soreness of the fauces, and frequently swelling of the lymphatic glands of the neck; but that, in a vast number, not a single instance was observed in which nodes were an attendant upon this eruption: 3d. That the ulcer with elevated edges, in the few instances, in which I had an opportunity of tracing it to its constitutional symptoms, was followed by a pustular eruption, which terminated in mild ulcers, pains in the joints, and ulcers in the throat, but no appearance of nodes; yet," as he states with exemplary candour, "that the instances in which I had an opportunity of witnessing distinctly the connexion between the primary and secondary symptoms of this poison, were too few to form a decided conclusion with respect to this particular: 4th. That the phagedenic and sloughing ulcers are generally attended by constitutional symptoms of peculiar obstinacy and malignancy, *viz.* pustular spots and tubercles, which form ulcers that spread in general with a phagedenic edge, and heal from the centre; extensive ulceration of the fauces, particularly of the back of the pharynx, obstinate pains of the knees and other joints, while nodes are frequently present, and the bones of the nose are frequently affected: and 5th. That, when an eruption, no matter what its character may be, is on a surface opposed by another, as on the fossa of the nates, upper part of the inside of the thighs, or in the axilla, the spots, if they do not ulcerate, extend into soft, moist elevations of the cutis, which ought to be treated according to the nature of the disease to which they belong. Thus, if they are syphilitic, with mercury; or, if papular, pustular, or tubercular, with the remedies recommended for the specific disorder. According to the established practice, these condylomatous swellings, as they are called, are universally treated with mercury; but I have, in innumerable instances, cured them and the other symptoms with which they are accompanied, without the exhibition of a particle of that medicine."

Mr. Carmichael, being now fully aware that the true Hunterian chancre will heal without the aid of mercury, thinks the propriety may be questioned of exhibiting that medicine for its cure; but he regards it as being of so very indolent a nature, and the surrounding parts so very slow in dispersing where mercury has not been employed, that, notwithstanding his conviction of the fact above admitted, he always directs mercury for such cases, where there is nothing in the patient's constitution to forbid its exhibition, and he continues its use as long as the callosity remains. Primary ulcers of this character now very seldom occur, and of course he does not often require to prescribe the medicine for their cure: he never orders mercury for the primary phagedenic and sloughing ulcers, from the most ample experience of their effects. With respect to all other primary ulcers, he never administers it for them, except they continue long, obstinate, and chronic, without shewing any disposition to heal. In such instances, he usually gives some preparation of mercury in alterative doses, watching closely its effects on the sores: if found injurious, its use is instantly discontinued. When the sores appear obstinate after the third or fourth week, he treats them with mercury, for the purpose of altering the actions of the part, as in cases of ill-conditioned ulcers of the leg or other parts of the body. Mr. C. from long experience, has not found that constitutional symptoms more frequently follow this plan, than where full courses of mercury had been employed from the commencement. He denounces it, as a sample of inconsistency and folly, to endeavour by mercury to prevent the accession of those constitutional symptoms, for which he should not exhibit that medicine, had they even actually made their appearance. The best and surest means of preventing the occurrence of constitutional symptoms are, in his apprehension, those which heal the primary sore with the least possible delay, and thus prevent the secretion of a morbid poison capable of contaminating the system.

Mr. C. in arranging under distinct heads the numerous appearances and symptoms produced by venereal complaints, professes to follow the same rule which has hitherto guided the judgment of nosologists, in classifying all other morbid poisons attended with eruptions. He thus regards the eruption as the most proper basis of the arrangement; and, without neglecting such auxiliary evidence as other attending symptoms may afford, considers them as of minor importance in determining the nature of the disease. By this method, he finds it easy to dispose of, in their appropriate places under the name of the eruption which belongs to their respective species, the numerous symptoms, both primary and constitutional, of venereal diseases which are so various, as seemingly to bid defiance to any attempt at arrangement. Next to the eruption itself as a symptom, Mr. C. regards the primary ulcer, whose characteristics are in general sufficiently distinct to enable us to foretell, with tolerable certainty, what will be the appearance of the eruption. These primary characters, however, may be destroyed by irritation, which often produces inflammation, and not unfrequently sphacelus; and, he says, not only the distinctive marks of the primary ulcer, but of the constitutional eruption also, may be so modified by the exhibition of mercury, where that medicine is improperly employed, as to become confused and indiscernible. Affections of the throat he holds to be too indistinct to afford any certain diagnosis; and, in forming our opinions, these appearances, he thinks, can only be esteemed as auxiliary to the characters of the eruption and of the primary ulcers. Still, however, they are not to be overlooked, because the affection of the throat which attends the papular eruption, and particularly if the disease has not been interrupted by mercury, is not an ulcerated, but rather an excoriated or erythematous appearance of the fauces, very similar to that which accompanies measles, small-pox, or trivial cases of scarlatina. The excavated ulcer of the tonsil was regarded by Mr. Hunter as the characteristic sign of syphilis in the throat; but our author has found it as frequently connected with the disease which follows the primary phagedenic ulcer; upon which also, he conceives caries and ulceration of the nasal bones, and extensive ulcers of the pharynx, to be frequently attendant; but, he says, the same appearances occur in those anomalous disorders that arise from derangement of the constitution, and are not of venereal origin. In every species of the disease, pains may affect the larger joints and other parts: Mr. C. therefore, holds them as a very equivocal symptom; he thinks it useful to know, however, that the shafts of the tibiæ are more frequently subject to this symptom in syphilis, and the knees in the phagedenic disease. Syphilis and the phagedenic disease are alike capable of occasioning nodes; these, for this reason, are equally equivocal and uncertain, as characteristic symptoms. These, and other observations present sufficient grounds, in our author's judgment, for adopting a radical change in the nomenclature of venereal diseases; he, therefore, proposes forming this on the characters of the eruptions, which afford the most certain criterion, whereby to distinguish one species from another. His classification, in consequence, is the following.

I. *The Papular;* or that form of venereal disease which is the most prevalent of all others, and is attended with the papular eruption.

II. *The Pustular;* or that which produces pustules terminating in ulcers, covered with thin crusts, and healing, like common sores, from their margins.

III. *The Phagedenic;* or that which is attended with spots having less of the pustular character than the preceding class, and frequently with tubercles ending in ulcers, covered with thick crusts, which extend with a phagedenic margin, and usually heal from their centre; the term is equally descriptive of the primary as of the secondary symptoms.

IV. *The Scaly;* or that which has the permanent scaly eruption: It is that disease, says Mr. C., which I have distinguished by the name of syphilis, but which has been extended by other writers to every description of venereal disease, although it is probable that every other was known in Europe before it. Anticipating objections to his arrangement, Mr. C. proceeds, p. 68, to obviate them, in these terms:—

"It may, however, be objected to the classification, that the nature of the disease cannot be known until the eruption takes place; and, on a loose computation, it may be regarded that nine cases out of ten of primary ulcers are not attended by constitutional symptoms; so that, in a great majority of cases, the disease has never arrived at the stage to which it is indebted for its name. To this objection I reply, that the primary ulcers afford a less decisive means of determining the nature of the disease than the secondary; yet, from their characters, when unaltered by irritation or mercury, we may discriminate their nature with sufficient certainty to decide on the precise eruption they would produce in their secondary state. For instance, 1st—the ulcer, without callosity, raised edges or phagedena, in fact, without any peculiar characters; and which may, therefore, be termed the simple venereal primary ulcer, produces the *papular* eruption which ends in desquamation; and the same effect is produced by a patchy excoriation of the glans and prepuce in men, and of the labia and vagina in women, and also by a gonorrhœa virulenta. 2d, The ulcer with raised edges produces the *pustules* which terminate in small ulcers covered with *thin* crusts, and which *heal from their margins.* 3d, The phagedenic and sloughing ulcers produce the pustular spots and tubercles which terminate in ulcers covered with thick crusts, which are accompanied with *phagedena, and heal in general from their centre.* 4th, The primary callous ulcer, or chancre, is attended with the well-known *scaly eruption, lepra* or *psoriasis.* In this concise view of venereal diseases," he concludes, "we may perceive that they do not form an exception, as is generally imagined, to that uniformity of symptoms and characters which marks the progress of all the other morbid poisons with which we are acquainted."

Before passing from this chapter, the principal doctrines of which we have here endeavoured to delineate, we would take occasion simply to remark, that the pathological theory unfolded in it is exceedingly ingenious, but perhaps too perfect to be susceptible of uniform confirmation by the results of practical experience, so liable at all times to be influenced by the circumstances of different and distant observers. We ourselves, indeed, in our limited sphere of observation, have more than once been distressingly puzzled by the occurrence of remarkable deviations from the laws of morbid action, by which Mr. C's classification is defined. Thus, for example, we have found in true venereal patients, during the progress of the disease's papular form, all the existing symptoms greatly exasperated by the spontaneous supervening of a dense crop of impetiginous pustules; and, in one case of the same papular kind, the person was seized with confluent small-pox, and between the groupes of this, had the original papulary patches distinctly remaining, not only throughout the variolous eruption, but during a tedious subsequent treatment. In the first instance, the new phenomena were in a great measure disregarded as not originating from a particular source, at least, so far as not to be allowed to withdraw attention from the primary affection. The latter proved a cause of extreme embarrassment, as the genuine venereal symptoms never appeared in any degree to subside, even when the system was threatened with being overpowered by the variolous disease. We have also known extensive phagedenic venereal ulcers become complicated with both the papular and pustular eruptions: and we are by no means certain but these, likewise, had a specific origin. We do not wish, however, to attach undue importance to these circumstances; but, regarded merely as forming an occasional incident, they are sufficiently perplexing, and do undoubtedly possess some claim on our consideration. Neither do we conceive that isolated facts of this kind can, in any way, affect the accuracy of Mr. C's. general conclusions; we mention them purely with a view to remind the respected author and our readers, of the difficulties opposed to every system of nosologic arrangement, by the apparently discordant features which disease is ever ready to assume; and, by consequence of the necessity of taking the first principles on which to found practical doctrines, from observations instituted under the direction of different inquirers, and subjected to the different influences of climate as well as of the very diversified relations of social life.

CHAP. III. *Papular Venereal Disease; comprising the complaints that are liable to be attended with an eruption of papulæ, which constitute the most simple, most easily cured, and least dangerous of all the forms of the disease.*—Mr. C. begins this part of his work with a description of the *primary symptoms* which, he says, are either: 1st, a simple ulcer, without induration, elevated edges or phagedena; but whose characters are not very remarkable; 2d, a patchy (this is a new but expressive term) excoriation of the glans and prepuce, attended with purulent discharge; and 3rdly, gonorrhœa virulenta. According to our experienced author, the ulcer, here denominated *primary*, has neither the indurated base of the true syphilitic chancre; nor the elevated edges that surround the primary ulcer of the pustular form of the disease; nor the phagedenic surface of the primary phagedenic ulcer. It is seldom observed at its commencement; but, in the few instances within Mr. C.'s experience, it exhibited a small pustule which continued one or two days on the external prepuce or body of the penis, and then formed a thin crust which, soon separating, exposed an excavated round, or oval ulcer, with a surrounding redness. This, in the second week, began to fill up, and afterwards rose above the surrounding skin, exhibiting a smooth surface, having the colour of a healthy sore, but without granulations, and having somewhat of a fungous appearance. The process of ulceration, as well as the surrounding redness, seems to cease as soon as the fungous stage commences; and this may exist an indefinite length of time. From three to six weeks, however, may be taken as the average period of the continuance of this ulcer from its commencement. This, like every other primary ulcer, is nevertheless exposed to be much modified by the patient's constitutional and personal circumstances. It is far more general than any other, and most frequently occurs on the glans and internal surface of the prepuce, where it is apt to induce phymosis. Sometimes, also, it is seated on the external surface of the prepuce, body of the penis and scrotum, in which last situation it is considerably raised, so as to resemble a soft wart; it is rapidly cured by an application of a strong solution of muriate of mercury, in the proportion of two or three grains to an ounce of water. In women, the labia, perineum, and fossa of the nates, are usually the seat of this kind of ulcer.

It is difficult or impossible, our author justly observes, to ascertain when phymosis has taken place, from what source the discharge proceeds, whether from these ulcers or from the patchy excoriation of the glans and prepuce; or whether it flow from the urethra and is a true gonorrhœa. He assures us, however, that it is frequently occasioned by these three affections at the same time; and argues from this circumstance, independent of other reasons, that they all arise from the same poison. This supposition, moreover, he regards as being almost confirmed by the fact, that the superficial ulcers, even when situated so far back as the scrotum or body of the penis, are in the majority of cases accompanied by one or both of the other two affections. In the excoriation of the glans and internal prepuce, the discharge, he adds, does not proceed from the entire surface, but from irregular, inflamed, excoriated patches, between which are interstices of sound cuticle; and from some observations of Dr. Munro and Mr. Whately on the similarity of appearance in the living membrane of the urethra and surface of the glans and prepuce, when secreting purulent matter, he is led to the opinion, that the discharge from these surfaces may be produced by the same poison, and is, therefore, in both instances, the same disease affecting different parts. That the ulcers in question and the excoriation of the glans and prepuce are frequently followed by the papular eruption, Mr. C. thinks every practitioner of moderate experience must admit, but, that gonorrhœa alone may also produce it, he holds to be more questionable. In support of this position, however, he states with his usual candour, that he has repeatedly seen men who asserted positively that they never had any primary symptoms except gonorrhœa; and many women also who, besides this discharge, did not exhibit any other primary symptoms; and affirmed at the same time that they never had primary sores. At first, he doubted the veracity of these people; but, having subsequently met with innumerable instances of the papular eruption in persons affected with gonorrhœa, combined with a purulent discharge from the glans and prepuce, a little consideration led him to conclude, that it was the same disease affecting different parts. Nevertheless, it must be admitted, he adds, that constitutional symptoms after a gonorrhœa is a very rare occurrence; and, therefore, it can only be esteemed as a natural, but not a very common consequence. As Mr. Hunter himself, who supposed that chancre and gonorrhœa arise from the same poison, affecting different surfaces, does not attempt to account for the infrequency of constitutional symptoms attending the latter, Mr. C., with a view of affording some clue to this hitherto unexplained and little-heeded fact, submits the following considerations.

"*First.* When we wish to inoculate the matter of other morbid poisons, the vaccine and variolous for instance, the earlier it is taken, and while the infection (the fluid in the vesicle or pustule, he means) is thin, limpid, and not purulent, the more certain are we of communicating infection.

"*Secondly.* Mucous surfaces, when assailed by contagious matter, are excited into a state of inflammation which rapidly passes into the suppurative stage.

"*Thirdly.* It may be inferred from the preceding premises, that inflammation and suppuration are the bars which nature opposes to the introduction of morbid poison into the system. These views will not only account for the infrequency of constitutional symptoms attending a gonorrhœa, but indicate a most important item in the laws which govern morbid poisons. It also adds another instance to the many already observed, of a vis medicatrix naturæ, or of those wise laws of the Creator, by which there appears an inherent power in the constitution of animals to resist injurious agents. The mucous surfaces, necessarily unprotected by cuticle, are more exposed than others to be assailed by a variety of morbid poisons; but they are more promptly than other textures excited to inflammation, which rapidly, or without breach of surface, passes on to the suppurative stage, which, as we are taught by our knowledge of the vaccine and variolous inoculation, renders a morbid poison innocuous. Hence we have strong reasons, from the above facts and considerations, to conclude that the rapidity with which mucous surfaces inflame and suppurate prevents the admission of morbid poisons into the constitution."

This train of reasoning is followed by a remark, to which we would attach some practical importance; it is this, in the author's own words, "the frequency of catarrh and influenza may not," says he, p. 79, "(under these views) be always occasioned by changes of atmospherical temperature, but may be often owing to contagious matter floating in the air; which is thus prevented from doing farther mischief than exciting a troublesome affection of the mucous membrane of the nostrils and branches of the bronchial tube." From this, Mr. C. proceeds to make extracts from Mr. Evans's work on Ulcerations of the Genitals,* which he justly observes, affords some important facts, that bear upon the obscure subject under consideration, and support the opinions he has ventured to offer. His remarks on the distinctive characters of chancre and gonorrhœa are stated by our author with less than his ordinary perspicuity; they conclude thus:

"In support of the opinion that chancre and gonorrhœa arise from distinct poisons, I may observe (although I will be accused of begging the question) that the constitutional eruption which follows chancre, is of the order of scaly diseases belonging to the species *lepra* or *psoriasis* of Willan, while that which attends gonorrhœa is papular, belonging to the species *lichen* of the same author."

Mr. C. informs us, that the buboes which occur in the papular form of the venereal disease, do not exhibit any particular characteristic by which they are distinguishable from those which arise from other venereal primary ulcers, except that they partake of the original mild character of the disease; and that, probably from the same cause, they are often remarkably indolent, occasioning no pain and but little inconvenience, unless by their tediousness. In all the instances but two, which occurred to him during a fifteen years' investigation of the subject, the foregoing primary symptoms were followed by a papular eruption, terminating in desquamation; in the two exceptions it was pustular, and can be regarded only as a slight deviation from a very general rule.

We come now to the *constitutional symptoms* of the *papular* venereal disease. The first of these is fever, attended with head-ache, pain in the shoulders and larger joints, and sometimes in the chest, with considerable dyspnœa, which ushers in an eruption that chiefly appears on the forehead, chest and back, but also extends in a more scattered way over the extremities. The fever does not subside on the appearance of

* The reader is referred to a long analytical notice of this excellent Book, in the No. of this Journal for June, 1820, p. 28—40.

the eruption, although it is at its height just previous to that event. It exists as long as fresh crops of the eruption continue to appear, and is usually accompanied with pains of the joints, which are most severe at night. The papulæ vary in colour from a pale red to a deep crimson: some of them are simply pimples, while others are almost advanced to the pustular state. The time of the eruption's appearance after infection is quite uncertain. Mr. C. has observed it to occur, in a few instances, in four or five weeks. In some patients, the papulæ are numerous on every part, but particularly on the face, back, and belly; in others, they are thinly scattered over the surface of the body. They do not all make their appearance together, as in the common exanthematous eruptions, but follow each other in succession; so that, on the same persons, some spots will appear, in their commencement, like small pimples with acuminated tops, containing pus or lymph; while others, on their decline, consist of exfoliations of the cuticle. In the latter stages, their colour becomes paler, and assumes a copper tint, while the exfoliation of the cuticle gives an appearance of scaliness, which is liable to be confounded with the scaly eruption of syphilis. They may, however, be readily distinguished from each other; for when the papular eruption is on the decline, and has assumed a pale red or copper colour, we shall find, on examining the patient, other spots in their papular or pustular form, which will point out the character of the eruption. The copper-coloured scaly surface of the declining papulæ, moreover, is more raised in the centre than its circumference, while the reverse is the case in the true scaly syphilitic eruption. As in all cutaneous eruptions, there is frequently a soreness of the throat; but this is widely different from that which takes place in syphilis, where there is a deep excavated ulceration of the tonsils, with little inflammation or difficulty of swallowing. In this disease, on the contrary, the soreness and difficulty of deglutition are considerable: and, on examination, the entire fauces, but more particularly the back of the pharynx, exhibit an erythematous appearance; not unfrequently also, there is considerable swelling of the tonsils, which assume, as they always do when swelled, an irregular appearance that is often mistaken for ulceration. The cervical glands, likewise often swell and ulcerate, but most frequently when the eruption is on the decline. These swellings are generally, but erroneously, esteemed scrofulous, on the supposition that the patient had scrofula lying latent in the system, which was brought into a state of activity by mercury; they are as liable to arise when this mineral is not, as when it is, exhibited.

After having wholly disappeared, the eruption will, in a few instances, return again and again, at uncertain intervals of from one to several weeks; each successive crop, however, will be less than the former, and attended with less constitutional derangement: these intervals are also greater as the disease subsides. If, however, the disease's progress has been interrupted by mercury before it has arrived at its latter stages, it is, by this means, rendered more obstinate and complicated. When that medicine is administered on the first appearance of the eruption, and while there exists considerable fever, with severe pains of the joints, requiring the lancet, the patient, is, in general, rendered much worse, his fever is increased, and the pains become more intense. On the other hand, if the mercury is postponed till the fever has subsided, the eruption will, in most instances, disappear under its use; and the pains, though not removed, will be alleviated. So soon, however, as the mercurial irritation has ceased, a fresh crop of papulæ will generally arise, together with an increase of pains, and, perhaps, soreness of the throat. Under these circumstances, another mercurial process is usually recurred to, and the yielding of symptoms, as in the first instance, induces a belief that the right path has been pursued, and that the former failure was owing to the employment of an insufficient quantity of mercury. The patient is, therefore, subjected to a more severe and protracted course of that medicine; and, while he is under its full influence, the symptoms often return with additional severity. This return of symptoms, says Mr. C. shews that the mercurial irritation on the constitution is no longer capable of suspending that of the poison, and this fact, he adds, is probably owing to the effects of habit. When the papular symptoms thus re-appear, the mercury should be discontinued, and the use of sarsaparilla, or other vegetable tonics, which, without weakening the constitution, promote the several secretions, be directed. Under this plan, if the patient's constitution has not been irreparably injured by mercury, he will, in general, have a surprisingly rapid recovery.

Mr. C. concludes his sketch of symptoms with a brief but faithful description of the venereal iritis. Intolerance of light, and defect of vision, he informs the inexperienced, are usually the first symptoms which induce the patient to seek advice. On examination, a want of the usual transparency of the aqueous humour is observable, and is caused by a deposition from the inflamed vessels of the iris; the pupilar edge of the iris, at the same time, exhibits a thickened and puckered appearance, which causes an irregularity in the circular form of the pupil, most observable at its upper part. A change of colour may be discerned in the iris, on comparing it with that of the other eye, if it has not also been affected, and although the conjunctiva partakes, in a slight degree, of the general inflammation of the eye, yet the vessels of the sclerotic coat, surrounding the cornea will be seen obviously enlarged, and deeply engaged in the inflammation, which is not strictly confined to the iris, but extends, more or less, to every part of the eye, as is readily evinced by the pain excited by pressure of the eye-ball. As the disease advances, adhesion takes place between the iris and capsule of the lens; the pupil becomes contracted, and portions of lymph not only plug up its orifice, but are deposited on the surface of the iris. Pus or lymph is seen lying at the bottom of the anterior chamber, the humours of the eye become more opaque, and vision more defective. The pain is sometimes intense and lancinating, but, in general, less considerable than might be expected from the extent of the disease affecting the delicate structure of the eye. Suppuration at length ensues, and, the eye becoming disorganized, the matter either makes its way through the sclerotic coat, or transparent cornea, but more frequently through the latter.

Mr. C. had stated, in a former work, that, with one single exception only, the eruption which accompanied venereal iritis, if any eruption was present, was the papular. His subsequent experience, which has been very great, enables him to confirm this statement, by declaring that, among the numerous cases of iritis which occurred during the latter period of his observation, "there was not a single instance of the disease accompanied by eruption, in which that eruption was not papular." Notwithstanding his uncommon experience, however, he still modestly, and with the candour of a mind "conscia recti," declines asserting that this venereal affection of the eyes is *only* attendant upon the *papular* eruption. This leads him to notice and to repel, with a just sense of injury, the disingenuous insinuations made, in reference to the subject of iritis, by a sordid egotist, who manifestly possesses neither pride nor principle. With this low being, however, it was altogether useless to reason: it had been enough, in our mind, to have left him to the bitter writhings which must arise from the reflection, that, though impudence and quackery may sometimes enable a worthless person to pilfer a knighthood, or even to acquire a large share of "filthy lucre," yet these shall never secure to any one the merit of pure virtue—a lasting and generous renown.

Our author considers the absence of nodes as one of the characters of the papular disease, but admits that, in a few cases, swellings occur over the tibiæ, which by some may be denominated nodes. They differ from nodes, however, in possessing much more of the inflammatory character, and in affecting obviously the integuments covering the bone, and not the bone itself, for they appear suddenly, and after continuing a few days, as rapidly disappear, without the exhibition of mercury.

Mr. C.'s treatment of the *primary symptoms*, consists in keeping the patient as quiet as possible, with very light diet, under every circumstance; but, if inflammation, with swelling and phymosis, be present, in strictly confining him to a recumbent position: in the latter case the antiphlogistic rules ought to be rigidly observed. The internal remedies he prescribes, are the vegetable and saline cathartics, with antimonial preparations. Unless the ulcers are very inconsiderable indeed, the gentlest exercise will disturb them; when they are at all irritable, confinement to the house is indispensable. These ulcers will heal under the use of any simple astringent wash or mild ointment: if, however, they pass into an indolent chronic state, mercury, in alterative doses, will certainly hasten their cure, by exciting a new action in the part. Mr. C. employs such local applications as are most likely to heal the ulcers as soon as possible; on the principle that the sooner an ulcer which secretes a morbid poison capable of infecting the constitution, is healed, the more likely is the constitution to escape contamination. Being satisfied of the fact, that the earlier the stage of the ulcer, the more active and virulent is its poison, and that, in proportion as it advances to the secretion of pus in place of lymph, its contagious properties diminish, he instantly endeavours to destroy the entire surface of such an ulcer, in its first stage, while it is yet excavated and secreting lymph, by a free application of lunar caustic; and, when the eschar separates, he usually finds a simple, instead of a poisonous sore, which is afterwards covered with lint moistened in a gradually weakened solution of nitrate of silver. He has not, by any means, found bubo to be apt to follow such applications of caustic to primary ulcers, if it be applied at an early period, when they are in their excavated state, secreting lymph, and of small extent.

When, in the second stage of a sore, its surface is either on a level with the surrounding skin, or raised above it, and pus instead of lymph is secreted, no advantage whatever shall be derived from applying caustic with the object of cutting off infection, as Mr. C. terms it, or altering its nature; the ulcer, in such case, has already attained much of the character of a common sore, and may be readily healed with a weak solution of the nitrate of silver, or the black or yellow mercurial washes. When these ulcers are much raised, and have a fungous appearance, touching them daily with sulphate of copper, hastens their cicatrization: for sores on the external prepuce or body of the penis, ointments, as that of zinc, either alone, or blended with a third or fourth part of the nitrated mercurial, are the best applications. Besides confinement to the recumbent posture, a patient, having a disposition to phymosis, should frequently inject warm water between the glans and prepuce; or poultices of bread and water may be applied to the entire penis, and the antimonial solution given in slightly nauseating doses. When, however, the inflammation is violent, and the penis considerably swollen and painful, if the most active measures are not immediately adopted, the inflamed organ will fall into a state of mortification, the symptomatic fever increase, the pulse rise to 110—130, and the patient be distressed with great thirst and restlessness. For all this, general blood-letting is urgently indicated, and should be repeated every six or eight hours, till the symptoms begin to yield. Leeching is scarcely admissible in cases of this kind; for, if the venereal discharge should come in contact with the wounds made by the animals, very troublesome sores may ensue. Sometimes a portion of the prepuce sloughs in such a manner, as to leave an opening through which the glans penis passes out, and the remainder of the prepuce lies behind, or at the frenal side of the glans, forming an useless appendage when the parts are healed. Mr. C. does not say how this is to be remedied: we have seen the "useless appendage" removed by ligature, and also by the scalpel, to the great comfort of the patient. When matter forms under the ligament of the penis, the pain is excessive; the entire organ acquires a state of constant tension, and becomes indurated in an extraordinary manner; and the integuments assume that red colour which indicates the presence of matter underneath, but no fluctuation can be observed, on account of the thickness and unyielding nature of the investing ligament; this circumstance can only be discovered by the previous symptoms. At length, the matter makes its way through that part of the dorsum penis nearest the pubis, and a probe will freely pass into the small round opening through which it flows. Sometimes it points over or above the pubes, and then always produces a deep abscess: occasionally, as the disease advances, the ligamentous covering of the penis ulcerates and sloughs, leaving a large foul ulcer with everted edges, on the dorsum. For preventing this, and its destructive consequences, a free incision of the dorsum, longitudinally through the ligament, before ulceration takes place, is the surest practice; and, so soon as the discharge shall have obtained a ready issue, all the symptoms will gradually give way, under the use of emollient applications. Sometimes, also, the matter finds a passage at the corona glandis: and a probe will pass from the aperture under the ligament, as far as the pubes; an opening at the upper end of this sinus, will contribute much to its obliteration: when, however, this does not succeed, the insertion of a seton will, in many instances, remove the disease, and leave the parts uninjured.

When warts follow the simple venereal primary ulcer, Mr. C. prefers to mercury or the common caustics, the application of *acetic acid* for their removal; and he declares that he has never known it to fail, in any instance. He recommends it chiefly for large warts with broad bases, on which it should be rubbed daily, by means of lint on the end of a probe; he cuts off those having narrow necks, and applies lunar caustic to stop the oozing of blood. The chancrous, or patchy excoriation is the most easily cured of all the venereal complaints; any mild astringent lotion, as the yellow mercurial wash, weak solutions of acetate of lead, or sulphate of zinc, injected five or six times daily, between the glans and prepuce, or even simple ablution of the parts, will heal the sore in a few days. "It is so easily cured, even under common attentions to cleanliness, that I cannot conceive," says Mr. C. emphatically "how some practitioners can have the conscience to subject their patients to a five or six weeks course of mercury, for a complaint that simple water may remove in a few days."

Gonorrhœa so very generally accompanies the patchy excoriation just described, that the appellation of external gonorrhœa has been given to it by many surgeons. Virulent gonorrhœa, in its early or inflammatory stage, is characterized by the nature of the discharge; this, though purulent, is thin, and stains the linen of patients with a greenish hue. During this period, also, which corresponds with the first stage of the simple primary ulcer in its excavated state, and when secreting thin ichorous matter, the disease is most virulent and infectious: as, however, the specific inflammation of the mucous membrane of the urethra subsides, the secretion from it becomes thicker, more purulent, and less infectious. At first, nothing further can be done, than to lessen the inflammation; but, when this runs so high as to excite sympathetic fever, general blood-letting sometimes, though rarely, is required.

In this stage, Mr. C. usually has recourse to solutions of tartarized antimony, with or without the magnesian sulphate. This medicine, says our experienced author, prevents the patient from indulging a good appetite, lessens inflammation, and is the best means for securing him from painful erections. During its exhibition, large diluent draughts are very beneficial; these, by causing frequent passage of the urine, wash off, without irritation, the virulent matter secreted by the urethra.

Mr. C. does not dispute the fact of almost instantaneous cures being effected, in the first stage of gonorrhœa, by injecting a strong solution of the nitrate of silver, yet he regards the practice as being attended with such risk of exciting severe inflammation of the entire urethra and bladder, and all the immediate, as well as secondary, train of evils connected with this calamity, that he has no hesitation in saying, that it is a practice which cannot be too strongly deprecated.

In the second stage of gonorrhœa, the discharge is thicker and more purulent, and the disease gradually loses its specific or infectious properties. At length, the purulent discharge ceases altogether, and the urethra either regains its healthy state, or has the increased mucous secretion, which constitutes gleet. At this period, such medicines are beneficial, as possess most power over the altered secretions of the mucous membrane, in all parts of the body; there is no necessity for their being endowed with specific or anti-venereal qualities. Than all others, the terebinthinates are the best, and the balsam of copaiba is the remedy upon which, with justice, the greatest reliance is placed; it should be given in as large doses as the stomach can bear. Cubebs, in some instances of Mr. C's experience, has answered his expectations; but, in the greater number, he has met with disappointment. When the discharge resists this treatment, mild astringent injections may be employed; but, when much irritability of the urethra prevails, even these are inadmissible: it is far better, says our author, wisely, to trust to time and internal remedies, than to tamper with stimulating applications, which may irritate the parts in their state of increased sensibility. He ascribes the inflammation of the testes and neck of the bladder, and strictures of the urethra, rather to the irritation of gonorrhœal matter, than to the effects of astringent injections, and, therefore, recommends these, as the best means of putting as speedy an end as possible to the cause of such painful visitations. When buboes accompany any of the primary symptoms, he has not learnt, from experience, that mercurial frictions will discuss them; on the contrary, the trials he has made, incline him to believe that this remedy tends rather to increase their inflammation, and, consequently, their aptness to suppurate. Leeches and cold lotions, with rest and temperance, will often disperse them; and, even under suppuration, they will heal much more readily when exempted from mercurial excitement. In this form of the disease, however, the bubo is often remarkably hard and indolent; and, in such cases, Mr. C. advises the repeated application of blisters, which cause either the dispersion or suppuration of the tumour. When a bubo is intolerably painful, he opens it with a lancet; in other circumstances, it is left to a spontaneous discharge.

We have now brought the reader to Mr. C.'s treatment of the *constitutional symptoms*.—The papular eruption and its accompaniments, he firmly believes, will yield, in every instance, to the powers of the constitution; but, to effect this, it sometimes requires several months, as the eruption may disappear, and frequently recur in successive crops. When the disorder is on the decline, its cure will be considerably hastened by alterative doses of mercury. Feverishness and pains in the joints, with dyspnœa and uneasiness in the chest, usually usher in the eruption, and require repeated abstractions of blood, which always bring great relief: at the same time, the other antiphlogistic remedies should not be neglected, and particularly the exhibition of antimonials. With these, Mr. C. usually combines the decoction of sarsaparilla, after the inflammatory action has been overcome; and, with this treatment, he seldom fails to remove the symptoms: when these do not yield readily, he directs pills of antimony and calomel with the decoction of the woods, in small alterative doses, which can be exhibited with safety; and have, in no instance, disappointed him in curing this papular form of the disease, the simplest indeed, he says, and most manageable of these diversified complaints. He presumes, in fine, that the ease with which this disorder is cured, in some degree arises from the fever or reaction of the system by which it is attended; and thinks that, in this respect, it bears a close analogy to the exanthematous poisons which the constitution overcomes by its own unassisted powers. Should we not, therefore, be careful, he asks, not unnecessarily to interfere with the powers of the constitution, which we have now sufficient evidence to assure us, are in every *instance* adequate to overcome the poison which induces this form of venereal disease? If the term exanthem, he continues, is intended by nosologists, to designate "a contagious disease, beginning with fever and followed by an eruption on the skin,"

the various forms of the venereal disease, but in particular the one under consideration, have as strong a claim to be included in the class, as either small-pox or measles. With such views, he states decidedly that venereal diseases *ought* to be treated on the same principle; and that, when we quit the plain dictates of pathology to follow those of mysticism, if not of empiricism, we only entangle ourselves in inextricable errors, rashly driving the eruption from the surface, regardless of the axiom of the philosophic Jenner, THAT ERUPTIONS ON THE SKIN ARE THE SAFEGUARDS OF THE CONSTITUTION.

Mr. C. asserts that at least three-fourths of all the venereal complaints which prevail in Britain and Ireland, are of that kind which gives birth to the papular eruption. This, he often repeats, and seems anxious to impress on the reader's attention, he is always certain of seeing yield to his mode of treatment, unassisted by the disgusting and injurious process of a full mercurial course. He advises confinement generally, but inculcates it as indispensable in cold weather. In proof of its utility, he states the rapid amendment of hospital patients. If such subjects, *cœteris paribus*, are really more readily cured than others, it would have been acceptable to see the facts on which this doctrine rests. In the papular form of the affection he is always better satisfied of the permanent safety of his patient, *when mercury has not been employed;* and he affirms that, if the eruption and its accompanying symptoms do not return in a few weeks, he may be considered as cured; but, on the contrary, *if he has employed mercury*, the disorder may return after many months, unless the medicine was exhibited when the disease was on the decline. He, therefore always, (it is ever *always* with our generous enthusiast,) considers a patient who has been treated *without* mercury, as being by much the most secure against a relapse. In many hundred cases of the papular eruption, he has not met with a *single instance* in which it was attended with nodes; our own experience leads to the same result nearly,—but we suspect the circumstance falls greatly short of being universal. Mercury, he concludes, should never be exhibited, unless at a late period after the disease has nearly yielded to the powers of the constitution; because it merely suspends the influence of the poison for a time, but does not supersede its action altogether, as is too certainly evinced by a return of the symptoms as soon as the mercurial action subsides.

"In fine," he says, p. 122, "our object in the treatment of the constitutional disease under consideration, should be,—1st, to moderate the action of the system, if the fever which attends and accompanies the eruption, should be violent: 2d, after the fever is considerably lessened or subdued, the exhibition of sarsaparilla, either alone or combined with antimonials, affords the most safe and efficacious mode of treatment. The action of sarsaparilla, particularly when assisted by antimonials, is to increase all the secretions; and these must not be checked, particularly that of the skin, by imprudent exposure to cold. The diet of the patient may be light and nourishing, but not heating or stimulating; and he ought to increase the quantity of mild diluting drinks he is in the habit of taking, which will assist the action of sarsaparilla * on the skin and kidneys; 3dly, when the eruption has declined, no new spots appearing, and those remaining all desquamating or scaly, while the patient still complains of lingering pains in his head, elbows, hips or knees, the disease being obviously on the wane, it may now be subdued altogether by alterative doses of mercury conjoined with antimony; and with this, the sarsaparilla may still be continued, either in the form of decoction or infusion."

Mr. C. next relates eight cases in illustration of the papular form of venereal disease, and of his mode of treating it;—on these, however, we cannot dwell. At p. 133, he returns to the subject of Iritis, on which he advances some excellent remarks. He assures us, that one good effect of treating venereal complaints without mercury is, that we are enabled to remove that accumulation of doubts, which are eternally arising while that medicine is employed, and which so often embarrass us in deciding whether we ought to attribute the occurrence of new symptoms to the disease or to the remedy;—to the *morbid* or to the *mineral* poison. It is his decided opinion, that iritis is frequently found in those who have never used mercury; and he believes it to be equally certain, that it has never been observed in a person who was salivated for any disease that was not venereal. Inflammation of the iris, whether it originates from venereal infection or any other cause, will readily yield to mercury, and the antiphlogistic remedies; because the mercurial action is one of the most effectual means of arresting the disorganizing process of adhesive inflammation, whether of the iris or of any other texture of the body; it, in fact, extinguishes the inflammatory principle, prevents the deposition of coagulable lymph, and promotes its absorption, when this has commenced; and thus affords an explanation of a seeming anomaly:

* The red sarsaparilla has been lately introduced into practice, and is far superior to every other description. Mr. C, gives the following formula for preparing the infusion in lime-water: R. Sarsaparillæ Rad. incisæ, ℥iv. Glycyrrhizæ Rad. contusæ, ℥i. Aquæ Calcis, lb. iv. macera per horas xxiv. in vase lentè clauso, dein cola.

that the symptom of a disease should be cured by a medicine which is incapable of arresting the disease itself.

Mr. C. appears willing to admit, on the authority of Dr. Thomson, that mercury, though a powerful auxiliary in the cure of venereal iritis, is not absolutely necessary to its removal. Dr. T. continues successfully to prescribe bleeding, blistering, and the antiphlogistic plan of treatment, carried to the utmost extent: in his own practice, Mr. C. throws in mercury as speedily as possible, with a view of stopping the adhesive inflammation; but, at the same time, he does not neglect to employ both local and general bleeding, blistering, the use of belladonna, and the antiphlogistic regimen. From many hundred cases, in which this method of cure was adopted, he has been led to these conclusions; 1st, that iritis is an attendant upon the papular eruption; 2d, that the eruption will occur after either the alterative or full courses of mercury; 3d, that gonorrhœa alone is sufficient to produce the papular eruption; 4th, that iritis will occur where little mercury, or none at all, has been employed; and, therefore, that the disease cannot be attributed to that medicine; 5th, that there is great utility in combining the depleting, with the mercurial plan; but the mercury must be discontinued as soon as the mouth is affected; and 6th, that, in a considerable number of cases, the iritis was not attended by any eruption whatever, and it yielded to the same plan of treatment.

With some very sensible remarks, interspersed with original observations, on the views of Mr. John Hunter, Mr. Abernethy, Dr. Adams, and Dr. Bateman, on the existence and frequency of diseases resembling, but really differing from true syphilis, our author brings this important chapter to a conclusion. He trusts that, in his account of the first class of venereal maladies, he has adduced sufficient evidence to satisfy any reasonable mind, that the same virus may produce the three primary affections he has described,—that these primary affections are all liable to be followed by the same train of constitutional ailments; and that all the symptoms of this, the *papular* disease, both primary and constitutional, may be easily recognized and distinguished as to their external characters, by those who endeavour to discriminate one disease from another. He also trusts, that he has offered strong grounds to infer, that the appearance of the venereal eruptions is far from irregular; but, that the embarrassment of the practitioner has arisen, not from any want of uniformity in the *effects* of those poisons, but in the circumstance of his ascribing to *one*, the diseases which arise from *several* poisons. The papular, pustular, and phagedenic venereal affections have hitherto been attended with more danger, he thinks, than the scaly or true syphilis; not, however, because they are, from their nature, in reality more formidable, but because they have been confounded with that disease, and consequently subjected to an inappropriate remedy. Thus it happens, he says, that the symptoms of those disorders recurring after the use of mercury, that medicine is again resorted to, and the mercurial irritation is alternated so often with that of the disease, that the patient at length falls a victim to their combined effects; and, in this way, numbers are annually destroyed. When once known and distinguished from syphilis, they are no longer troublesome: the powers of the constitution, assisted by simple remedies, are sufficient for their cure; they may be tedious, but they cannot be destructive.

Chap. IV. *Pustular Venereal Disease.*—In this, the *primary ulcer* has a reddish-brown surface, bordering closely on the phagedenic character. It begins in the shape of a small pustule attended with itchiness, and has elevated, well defined margins: it is not excavated, however; but is either level with the surrounding skin, or considerably raised above it: in general, it has a chronic nature, and evinces little disposition to spread. Its well defined and elevated edges, with the absence of smoothness and all other fungous appearances, distinguish this from the primary ulcer of the *papular* disease: its well defined margins, its surface not being irregular and "corroded-like," and also its exemption from painful and acute symptoms, distinguish this from the phagedenic primary ulcer: and its want of the callous edge and base distinguishes it from chancre. Ulcers of this kind are most frequently found on the external surface of the prepuce, the body of the penis, and occasionally on the scrotum, and vary from the size of the smallest pea to that of a shilling. When of the former size, they often form a circle round the orifice of the prepuce, and occasion, when the sores heal, a permanent phymosis which can only be removed by the knife.

Mr. Evans, in his work on ulcerations of the genitals, had stated his conception of our author having been led into error, in his first edition, when he supposed that the primary ulcers of the papular and pustular forms of the disease are essentially different: Mr. C. however, repeats his entire conviction of the accuracy of his opinion, and here recapitulates the chief circumstances which, he conceives, sufficiently warrant him in the assumption, that these ulcers are essentially different, and arise from different poisons: nevertheless, he admits that he has not met with many instances in which he was enabled to follow this (*the pustular*) ulcer to its constitutional symptoms. From this discrepancy of sentiment entertained by two gentlemen of high talents, and the greatest professional experience, regarding a matter of almost pure observation, we would deduce, at least, this instructive lesson. That too much circumspection can never be employed in drawing the general conclusions on which it may be proposed to rest the foundations of an important modification of practice.

Mr. C. has had occasion frequently to observe that the buboes, which proceed from the pustular virus, resemble the primary ulcers of the same specific infection, in their tendency to form projecting or undermined edges, particularly where much mercury has been employed; and, if these edges are not removed by art, they remain for months, and perhaps years, without healing. He always uses the scalpel for their removal, and thus, generally shortens their treatment. Full courses of mercury universally increase their tendency to burrow; but, he says, the ulcer is too often attributed altogether to the disease; and, the more it spreads, the more abundantly is the infallible *specific* administered. In the pustular *primary* ulcer, stimulating and caustic applications certainly produce no beneficial effect; and, if the sore is irritable, encourage it to extend. Our principal care, he adds, should be to keep the patient at perfect rest, which, with gentle astringent applications and mild ointments, seems to be all that is requisite: his treatment of the *constitutional* symptoms, is little different from that of the constitutional symptoms of the phagedenic ulcer, at the consideration of which we shall forthwith arrive.

Passing some cases which, of course, go to prove the supremacy of the practice they are adduced to exemplify, we come to the following remarks on eruptive diseases, with the transcription of which we shall terminate our view of this chapter.

"All eruptions, venereal or not venereal," says Mr. C. p. 160, "imperceptibly glide into those of the nearest character; and, it often happens, that practitioners can only determine the nature of the eruption, for which he is called upon to prescribe, by an attentive consideration of its progress. Thus, the chicken-pox, which is a vesicular eruption, is often found to contain pustules so large and so closely resembling those of small-pox, that it is only by attending to the progress of the eruption, and perhaps its termination, that the one can be distinguished from the other. On the contrary, small-pox often exhibits so many papulæ and vesicles, or half-formed pustules, that the character of the disease is not very easily distinguished, even by the most experienced practitioner, until its progress determines its nature. The common itch exhibits three orders of eruptions at the same time: *viz.* pustules, vesicles, and papulæ; and yet the venereal character of the disease is so obvious, that almost any person can, without hesitation, decide upon its nature. In the same manner, venereal eruptions are sometimes observed to glide into those of the nearest character; thus, the papular eruption may exhibit a few pustules which, like the pustular venereal eruption, form their crusts instead of ending in desquamation; but still the character of the disease is so apparent, that there is not, by any means, the same degree of ambiguity which attends the variolous and varicellous diseases; and in the same way the pustular disease may exhibit papulæ among its pustules, to which the same observations may be applied; but this practical one is worth adding, viz. *that the more closely the eruption approaches to the form of papulæ, terminating in desquamation, so much the more mild, yielding, and manageable will the disorder be found.*

"The phlyzacious pustules, which have been observed to arise from the ulcer with elevated edges, all terminate in superficial sores, covered with thin scaly crusts; yet, although a few pimples may be intermixed with these pustules, the general character of the eruption is sufficiently obvious to evince its pustular nature; and the general mildness of the eruption and of the ulcers which it produces, compared with the thick crusts and spreading ulcers, with phagedenic margins of the phagedenic disease, sufficiently points out the distinction between them. The eruption which I attribute to the ulcer with elevated edge often exhibits at the same time on the same individual new formed pustules; others in their scabbing state, with an intermixture of small ulcers whose crusts have fallen off, and of discoloured patches of skin when they have healed, so that nothing can be more disgusting than the appearance of a patient under those circumstances. In this state of the surface there is nothing more efficacious for clearing the skin of all this foulness than sulphur fumigations. Baths, impregnated with sulphuretted kali, (*potassa sulphuretum, P. L. and E.*) and the nitro-muriatic acid baths, are also useful, but not equally efficacious with the sulphur fumigations. Smearing the body with equal parts of tar and sulphur ointments is also of service; with these applications I also combine the internal exhibition of sarsaparilla and antimonials.—The pustular venereal disease, p. 164, both in its primary and secondary symptoms, forms the natural link between the papular and phagedenic venereal diseases. It is more obstinate and inveterate than the papular, but in no instance possesses the malignity and destructive tendency of the phagedenic: the reader need only contrast its symptoms with those of the papular disease, to observe the wide distinction between them."

Chap. V.—*Phagedenic Venereal Disease.* Mr. C. proposes, in this part of his essay, to describe the phagedenic and sloughing ulcer, and their constitutional symptoms. The phagedenic has an appearance of local corrosion, and neither exhibits granulations nor surrounding induration. Sometimes, it spreads with rapidity, causing great destruction of parts in a few days; in other instances, it creeps on slowly, healing in one place and making progress in another: unlike a chancre, however, instead of being checked by mercury, it is almost always rendered more inveterate and rapid in its progress by that mineral. It attacks the glans penis more frequently than any other part, but usually proceeds to affect the prepuce, which it often entirely consumes; and, continuing its depredations on the corona and glans, at last effects their total destruction. When this takes place, the ulceration usually receives a sudden and permanent check: at other times, a spontaneous arterial hæmorrhage occasions a favourable change. This hæmorrhage is often very profuse; and, in many cases, requires to be restrained by a ligature: it is an occurrence, however, that is in general fortunate, for in such cases the ulceration is often stopped in its progress by this cause alone. It happens, also, but more rarely, that, notwithstanding every anodyne and lenient application, the ulceration will gradually proceed, until the entire penis be destroyed. There is another circumstance characteristic of this ulcer, worthy, says Mr. C. of remark, *viz.* the frequent return of ulceration, after the part has healed, to the very same spot which was first affected.

Notwithstanding, Mr. C. has treated a very great number of cases of the phagedenic ulcer, he has never yet, he says, had an opportunity of seeing it at its commencement: he is, therefore, still ignorant whether it arises from a pimple, pustule, or vesicle; or whether it passes immediately from its origin into the phagedenic state. On our own experience, we would venture to say, that it may and does originate indifferently from any one of these sources: we think we have known it more than once proceed from a pimple, a pustule, a vesicle, a mere abrasion, or even in the course of a single night, from a part in which nothing more than a slight sense of tingling and itchiness, without discolouration, had been perceived. This peculiarity, in our opinion, is determined in a great measure, by the rapidity with which the constitution admits the particular taint whereon the nature and characteristic appearances of the phagedenic ulcer depend: though the primary ulcer, and other symptoms usually come first under observation, we entertain no doubt whatever of their receiving their distinctive modifications from those of the constitutional disease. Our experienced author has observed, in two or three instances, as we also have done, that before the ulcer decidedly assumes the phagedenic character, it appears as a small excavated sore, covered with white adhesive matter; and he thinks it probable that, in this state, by the application of escharotics, it is as capable of being healed as any other primary venereal ulcer, before it passes into the phagedenic state.

Surgeons have seldom an opportunity of observing the *sloughing* ulcer in its earliest stage: generally, it gives little uneasiness, and thus passes unheeded until it has existed for several days: it is more untractable and destructive than the phagedenic. A minute black spot, resembling a grain of shot, in colour as well as size, is its first appearance; this, however, if examined by a practised eye, will be recognised as a mortifying slough, extending to some depth below the surface. Sometimes, this slough will increase to only three or four times its original extent; and at others, until it engages a considerable portion of the penis, before a line of separation can be discerned between the living and the mortified parts. When the separation at length takes place, we do not find a clean granulating sore, but an eroding phagedenic ulcer, which begins a new kind of depredation on the surrounding parts, equalling the virulence, but not the rapidity of the preceding sloughing process. Suddenly these parts are attacked by severe pain, and afterwards assume a bluish cast; and, on the following day, they are covered with a slough. In this way, the destructive malady extends its ravages, by alternate sloughing and ulceration, until, in the one sex, the entire penis, scrotum, perineum, and pubes are destroyed; and, in the other, until the labia, nymphæ, vagina, anus, nates, and even the bladder and uterus,* are engaged in one extended and malignant state of putrefaction. But if, as Celsus had also described, the ulcer is stopped in its progress, and a portion only of the penis destroyed, the orifice of the urethra becomes so contracted, that the urine passes with great difficulty, unless care is taken to preserve the passage open during the healing of the ulcer.

In most instances, the phagedenic and sloughing ulcers may be readily distinguished from all others, after they have assumed their peculiar characters: but the former does not immediately, on its first commencement, manifest its true phage-

* Mr. C. speaks conjecturally in regard to the precise state of these two last organs: Celsus says, without reserve, that the disease extends to the bladder; from dissection, we can assert that the uterus has been sphacelated in consequence of the spreading of the venereal phagedenic ulceration. Our author would have done a service to pathology, had he added to his valuable illustrations of successful practice, a selection of cases, wherein the morbid effects of the disease had been ascertained by inspection of the parts after death.—*Rev.*

denic nature. As occurs in the early stage of all morbid poisons, some difficulty may arise in distinguishing this from the other primary venereal ulcers. Our author, however, is of opinion, that it displays its peculiar symptoms so early, that embarrassment cannot often arise from confounding it with an ulcer, which becomes phagedenic from ulceration; for, he adds with examplary candour, "excessive constitutional irritability will cause a venereal, as it will cause any other, to become phagedenic, however originally mild in its nature." Young practitioners especially will, therefore, find great interest in attending to the progress of such ulcers, so as to ascertain, if possible, whether or not they assume the phagedenic character soon after their commencement. Some very judicious practical remarks, which we shall give in his own words, conclude Mr. C.'s description of these primary symptoms of the phagedenic venereal disease.

"These characters of the different primary venereal ulcers, already noticed," says he, p. 170, "are, I trust, sufficiently obvious to enable us, in a great majority of cases, to distinguish the commencement of diseases which take very different courses, and require, in many respects, different modes of treatment. Mercury improperly exhibited, neglect, irritation, and imprudence of various kinds, on the part of a patient, may so irritate and inflame an ulcer, as to render it impossible to decide on its character, until this adventitious inflammation is removed. The same difficulty may arise from the same cause, in enabling us to decide from appearances, whether a person has been inoculated with the vaccine or variolous matter, although nothing can be more distinct than the characters of their respective primary ulcers (if I may be allowed the term) when not thus affected. Inflammation thus occurring on primary venereal ulcers often excites mortification of the part; but then mortification or slough, thus produced is totally different from that which affects the venereal sloughing ulcer. In the former instance, when the sloughs separate, a clean granulating sore is exposed; and, in the other, a phagedenic surface, which proceeds alternately sloughing and ulcerating, as long as the disease continues. As far as my experience extends, buboes are less frequent in this form of venereal disease than in any other. Before ulceration, they do not exhibit any peculiar characters; but, afterwards, they partake of the general malignity of the disease. The edges of the ulcer become undermined, jagged, and irregular; and there is no chance of inducing them to cicatrize, until these projecting edges are removed, which may be done either with caustic or the knife;—the latter is the mode I always prefer, as the least painful and tedious."

Mr. C. distributes his account of the *constitutional* symptoms of the phagedenic and sloughing ulcers into four heads: in our condensed view of his doctrines, we shall adopt the same plan.

1st. The primary symptom is an eruption of tubercles, pustules, or spots of a particular tendency, which quickly degenerate into ulcers, covered with thick crusts; these ulcers usually heal, in a peculiar manner, from the centre, while, at the same time, they are extending at their circumference, with a phagedenic border. These are often preceded by a high degree of fever, which subsides, or is altogether removed, on the eruption being established. Sometimes the patient will complain of being generally unwell, for a considerable period before the eruption, without being able to state any particular symptoms to account for his indisposition; nevertheless, a general listlessness, pallid countenance, languid eye, broken rest, inappetency and feverishness, sufficiently evince the secret workings of disease. In other instances, acute nocturnal head-aches, with soreness of the scalp, or slight dyspnœa, with tenderness of the breast-bone upon pressure, afford the first indications of a venereal taint. These, symptoms, however, may usher in any of the other forms of venereal malady; but they are most severe, certainly, in the phagedenic.

Mr. C. holds these to be the more usual characteristic signs of this disease, that the crusts which form on the spots assume a conical figure, and that the ulcer generally heals at its centre, while it is spreading with a phagedenic margin; to this general rule, however, he claims the reasonable exception, that accidental circumstances may occasionally interfere, to produce deviations from the ordinary course. He gives an ingenious explanation, by his friend Mr. Farrel, of the circumstance of the ulcer healing in this manner, *viz.* that it may be owing to the destruction of the cutaneous structure, in its centre, and that the skin being there destroyed, the parts underneath throw out granulations, which soon cicatrize; while, at the same time, the ulcer makes progress at its circumference, in accordance with the pathological law, that diseased affections, as well as inflammation, extend in the direction of the tissues they first attack, leaving untouched other tissues in their immediate vicinity. If, therefore, he reasons, this peculiar mode of healing depends upon the destruction of the cutaneous tissue in the centre of the ulcer, it is obvious that, though a frequent occurrence, it does not constitute on indispensible characteristic distinction.

2nd. The ulceration of the throat which attends the phagedenic disease, is of a most formidable nature. It commences in the form of a small white aphthous-looking sore, which usually attacks the velum, or posterior part of the pharynx, but more frequently the latter; sometimes, but more rarely, it begins in other parts of the throat. If not checked, it rapidly engages all the pharynx, extends upwards into the nostrils, and is too often followed by caries and exfoliation of the spongy bones, with tenderness of the ossa nasi, with a foul discharge from the nostrils. In its progress towards the mouth, it also affects the tonsils, and seizing upon the velum and uvula, speedily destroys them; thus, on examination, the mouth exhibits one continuous ulcerated cavity, covered with white viscid matter, extending from the palate to the lower part of the pharynx. Should the disease, as is frequently the case, descend into the larynx, it brings the patient's life into the greatest danger, and is accompanied with a most distressing train of symptoms, *viz.* a whispering stridulous voice, constant cough, and copious expectoration of viscid matter, with restlessness, extreme anxiety of countenance, emaciation, night sweats, rapid pulse, and all the concomitants of pulmonary consumption. When the epiglottis has been partially or entirely ulcerated, it can no longer perform the office of a valve. Foreign bodies, in consequence of this, slip into the wind-pipe, as often as the patient attempts to swallow, and excite violent irritation, and fits of coughing. Mr. C. has known two instances of sudden death from this cause; but, in general, persons so affected linger many months, and at length die phthisical. He never yet, he assures us, witnessed a recovery when the larynx was decidedly ulcerated, although mercury, cicuta, opium, blisters, and a variety of other means were employed.

Ulceration of the nostrils may be suspected, when the breath becomes offensive, with an obstruction of breathing through the nose; it ceases to be doubtful, when a foul discharge comes from the nostrils; exfoliations of the bones, and a sinking in of the cartilage, will afterwards prove the disease's ascendancy. Mr. C. found the venereal affection of the nose to be an obvious symptom of this form of the affection, and could readily trace it, in most instances, to the phagedenic primary ulcer: he never saw a case of it conjoined with the papular eruption, or the scaly syphilitic lepra, or psoriasis; on all occasions in which an eruption was also present, this was of the pustular kind, and had either formed scabs or ulcers.

3rd. While the patient is suffering from the eruption, he is, likewise, in general, afflicted with severe and obstinate pains in the joints, particularly in the knees, wrists, and ankles, which often become swollen, red, and painful, under the slightest touch. Usually, also, the knee is affected with the swelling, and other symptoms that denote acute inflammation of the synovial membrane. Mr. C. regards this state of the knee as being of so frequent an occurrence that it may almost be esteemed as one of its peculiar characters.

4th. Nodes have never been seen by Mr. C. in any instance of the phagedenic venereal disease, except where mercury was previously administered; but he regards the question, whether they, as well as obstinate enlargement of the testes, do occur in this form, when mercury has not been exhibited, as still remaining undecided. Indolent swellings, containing a serous fluid, and many other anomalous symptoms may, and do supervene: our author, however, has not found them in any cases, except those where mercury had been largely employed; so that he says, we have as much reason to attribute them to the agency of the medicine as to that of the morbid poison, or, more probably, to the combined effects of both. This is rather a "novel" mode of philosophizing; at present, however, we are not critics, but simply analytical journalists, and shall, therefore, pass on from this, and also the remaining portion of the paragraph, p. 177-8, which contains more self-complacency and snappishness than to us appear quite befitting.

Mr. C. is satisfied, that every experienced practitioner will admit, that the group of constitutional symptoms he has described, do often resist reiterated courses of mercury; and that, even while the patient is under the fullest influence of that medicine, but most frequently afterwards, new symptoms, not before observed, will arise, and particularly nodes; but such, he acknowledges, is the apparent perversity of the disease, that after repeated courses of mercury have failed, the patient will sometimes recover under a new trial of the medicine; and *that*, he emphatically adds, at a period when his hopes and his strength have been nearly exhausted. Neither does he entertain any doubt, but that the obstinacy of this disease perpetually (*perpetually*?) arises from the premature and indiscreet interposal of mercury, which interrupts and encumbers its natural progress. The vulgar, he says, often make use of terms which have originated in a close observance of nature, and he cannot but refer to this source the very frequent expression, "*the disease has been set astray*" which is usually applied to the untoward symptoms that occur in small-pox, measles, and scarlet fever, when the eruption suddenly recedes: and, also, to the unexpected attacks of venereal secondary symptoms, when the constitution is under the influence of mercury. In the morbid poisons first mentioned, he proceeds, we know that, on the re-appearance of the eruption, those dangerous symptoms, which arose from the affection of some of the internal organs, and threatened the patient's life, immediately decline; and if we may recur to analogical reasoning, *(wherein does the analogy consist ?)* in aid of our experience, the propriety of being guided by those facts, in the treatment of the malady under consideration, will not be disputed. Such, then, are the grounds on which our zealous author claims being permitted to argue, that the mercury which intercepts the natural progress of this morbid poison, and often removes the eruption, and heals the constitutional ulcers of the skin and throat, *transfers the disease to the deep-seated parts—the periosteum, fasciæ, and bones;* and that this is *the true reason* that we so frequently witness the occurrence of nodes, and other affections of the deep-seated parts, after the patient has undergone the "fullest" influence of mercury, and flattered himself, in the disappearance of the disease from his skin, that it had been altogether subdued in his system. He conceives himself to be still farther supported in his conclusions, by what he holds to be a fact, that the bones are seldom or never affected in those who have not used mercury. Nor, he concludes, are we to forget, in the argument, our knowledge of the proper treatment of yaws, between the symptoms of which, and the phagedenic venereal disease, there exists the most striking similarity.

Mr. C. illustrates his treatment of the phagedenic ulcer, by eight cases, to which we can only refer: that numbered XXI. is very interesting. It will yield, he generally finds, to the means calculated to remove pain and inflammation: with him, these are, absolute rest in the recumbent position; venesection in proportion to the intensity of the febrile and other symptoms; antimonials, in nauseating doses; warm poultices of bread and water; warm fomentations, in the form of a stupe or injection, between the glans and prepuce; and opium, hyoscyamus, and cicuta, in anodyne and narcotic proportions. When the ulcer has been brought to excite but little uneasiness, solutions of nitrate of silver, or mercurial washes suit some cases; in others, no application seems to arrest its progress. In such instances, a spontaneous hæmorrhage, which is sometimes very profuse, often gives an immediate check to the disease. Our author has often derived the happiest results from imitating this "process of nature," *(this accident rather)* by paring off the irregular and jagged surfaces of the sore, and encouraging the bleeding, by immersing the parts in warm water. When the ulceration is extensive, irregular, and chronic, a band of integument connecting one part of it to another, occasions much irritation. This is to be divided, when rapid amendment will follow. When the frænum is seized in the same way, as it occasionally is, division of it with a bistoury ensures success. Venetian turpentine, and balsam of copaiba, in one or two parts of olive oil, and other stimulating applications, are extremely useful to many sloughing venereal ulcers, both on the penis and the groin; they interrupt the phagedenic process, and, in a few days, produce a clear granulating surface.

Mr. C. has not obtained any advantage from emollient or fermenting poultices, in this kind of ulcer; he finds the terebinthinate applications, or a mild camphorated tincture of myrrh more beneficial. They correct the fetor, and stimulate the sound parts to throw off the sloughs; but unfortunately, he says, have not the power of preventing their renewal. One might almost conclude, that the kind of vital action capable of inducing the rejection of one slough, should be sufficient to resist the occurrence of another; but there is no reasoning against facts. Change of air is decidedly advantageous, bark injurious, in this description of sore; which, moreover, not unfrequently improves under large doses of opium and hemlock. When such ulcers have gained considerable ground, an unfavourable issue is to be apprehended: when part of the prepuce or glans only is ulcerated, judicious management may be expected to retrieve the patient from his perilous situation. This brings us to the treatment of the *secondary* symptoms.

When the fever which ushers in the eruption, or accompanies it, runs high, and is accompanied with severe pains in the head, chest, and joints, general blood-letting may be necessary; and opening medicines, with antimonial diaphoretics, will be useful auxiliaries. Pain in the head sometimes readily yields to a blister on the occiput, or nape of the neck; this stage of the disorder requires, also, confinement to the room, with low diet and diluting drinks, as in the other exanthematous diseases. If, however, the fever be inconsiderable, or have been reduced, decoction or infusion of sarsaparilla, in such doses as the stomach will bear, conjoined with antimonials, may be continued till the patient recovers. Mr. C. has made many trials of the decoction of guaiacum-wood, combined with pills of gum guaiacum and antimonial powder, in this form and stage of the venereal disease, with considerable effect; but he places more reliance on the sarsaparilla. All these medicines increase the secretions; and, in cold weather, ought to be exhibited with great caution. In extensive irritable constitutional ulcers with phagedenic edges, we shall find much benefit in combining cicuta with the decoction of the woods; the hemlock may be given in powder or extract, and in such doses as shall sensibly determine its peculiar effects on the constitution. Mr. C. believes the nitrous acid to be particularly useful, when the cutaneous affection is characterized by tubercles, which spread into foul and extensive sores; these tumours recede under its use; but, either before or after ulceration, (*i. e.* at all times, as we suppose) it will not fail, when taken freely, to prove a most valuable remedy. When the patient's nights are disturbed by pains in the joints, and general restlessness, the compound powder of ipecacuan, in diaphoretic doses, is by far the best anodyne; and, if these pains are severe during the day also, there will be advantage in repeating this medicine every sixth or eighth hour, in conjunction with the sarsaparilla in place of the antimonial solution.

"Under this simple plan," says Mr. C. p. 201, "I have succeeded in bringing the most alarming cases, in the course of six, eight, or twelve weeks, to a favourable termination. But if mercury has been unnecessarily exhibited during this period, as many months, nay years, might have been required," *(your "Athenian" reviewers, Mr. C. will call this a prophetic assumption)* "to conquer a malady which is *chiefly* rendered formidable by injudicious interference, and which, too often, leads the victim of mal-practice, through a disgusting and offensive train of symptoms, to a painful and lingering death. Half measures will not answer. Thus, many, in other respects, judicious practitioners, adopt the plan of giving mercury in alterative doses, conjoined with sarsaparilla; but, although this is not productive of the same mischief as full courses of that medicine, *yet I have proof, the most positive, in numerous instances, that it can serve no other purpose, but to protract the disease.* I would not, however, be understood to exclude the use of mercury altogether, for the cure of this, the most formidable of venereal complaints. It is against the *abuse*, not the *use*, of this remedy I contend; for none can be more useful than this, if applied at the proper juncture; *and that juncture is, when the disease is on the wane, having, in a great degree, yielded to the powers of the constitution, assisted by the remedies already mentioned.* This stage of the disease will be indicated by the subsidence of acute fever, the healing of the constitutional ulcers, and the termination of such recent pustular or tubercular spots as occur, into a kind of *scabby desquamation*, instead of spreading into ulcers covered with thick crusts, like their precursors. Mercury will not then interrupt the natural progress of the disease, but rather hasten it to its termination with well-timed assistance, while the chance is reduced to little or nothing, of exciting any venereal affection *(venereal affection?)* in the deep-seated parts. Instead, therefore, of injuring the patient's constitution, perhaps irretrievably, by premature and repeated courses of this medicine, the advantage is inappreciable of withholding its application, till the season arrives when it may be administered with a well-founded prospect of success."

Mr. C. subjoins to these, what he denominates a plain practical observation; it is this, that for scaly eruptions, (no matter whether their scaliness has appeared in the very commencement, as in syphilis, or towards their termination, as in other venereal complaints, when the disease and the eruption are equally on the wane, and yielding to the powers of the constitution,) mercury may be exhibited with every prospect of advantage, or, at least, with little danger of inflicting the injury which is seen to occur if the spots, at the time, are destitute of the scaly appearance. Under these views, he regards the excitement of mercurial action in the system, as a chief indication; and, when the skin or throat is affected, prescribes the solution of muriated mercury, or the compound calomel pill, with the sarsaparilla decoction, on account of their tendency to affect the skin. For delicate persons, he directs the decoction, and five grains of blue pill every night; but if they have nodes, or affections of other deep-seated parts, when it may be necessary to excite a full mercurial action, for the purpose of influencing their "low organization," mercurial frictions are often preferable, from their being less likely to irritate the stomach or bowels.

Phagedenic ulcerations of the throat require prompt and decisive measures. When small, with a white "aphthous-looking" surface, our author treats them with oxymel æruginis, the solution of nitrated silver, or that of the oxy-muriate of mercury, applied frequently with a hair pencil, or other apt instrument; when extension, with partial or complete destruction of the uvula, and a great portion of the velum, he employs fumigations, with the red sulphuret of mercury, or the hydrargyrus cum cretâ, every fourth or sixth hour. He endeavours to reconcile the apparent inconsistency of this practice with his previous doctrines, by stating simply, that it is always better of two evils to choose the least; *i. e.* to use a remedy capable of checking a dangerous ulceration, even though that remedy may render the general disease more unmanageable. Unless when the ulceration has extended upwards into the nostrils, or downwards into the larynx, these fumigations fulfil the desired indication: when either of these misfortunes has supervened, it is difficult to decide on the means to be employed. Mr. C.'s method, and his reasons for its adoption, are the following. Having found the internal use of mercury disadvantageous, he prefers, in the first instance, nearly the same external applications of this remedy (mercurial fumigations) which were recommended for ulceration of the throat, but modified to meet the difference of structure and situation of the parts. With this object in view, he anoints the nostrils, morning and evening, with a liniment composed of one part of the nitrated mercurial ointment, to three of olive oil; and, at the same time frequently injects the weak yellow mercurial wash. If these means, aided by the decoction of sarsaparilla, do not succeed, the mercurial fumigations should be substituted; and, if even these fail of putting an end to the ulcerating process, a circumstance that will rarely occur, our author proceeds to give mercury internally, in addition to the external applications. He restricts the trial of these, however, to a few weeks, but carefully limits their use, so as not to excite salivation. When the nasal bones become affected, they must, of course, exfoliate before the parts can possibly heal. Mr. C. advises sending persons, in such a condition, to the country, where they should live chiefly on a vegetable diet and in well-ventilated apartments.

Phagedenic venereal ulceration of the larynx has peculiar symptoms; these are, in chief, hoarseness, an increasing croupy cough, anxiety of countenance, expectoration of thick viscid phlegm, progressive acceleration of the pulse, night sweats, emaciation; and, indeed, all the characteristics of pulmonary consumption. Our author has found reason to believe, that this ulceration commences in the pharynx, and extends from this to the epiglottis, and afterwards the larynx usually becomes affected. When this last part has been implicated, direct applications, he says, are out of the question; fumigations or solutions of nitrated silver, may be applied to the epiglottis; to this, however, they cannot. His only resource, then, consists in counter-stimulants applied to the skin, and the internal use of mercury. Blisters, he is satisfied from experience, are not merely useless; they excite unnecessary pain, and are really injurious; caustic issues over the thyroid cartilage sometimes afford a little relief; and, in one instance, moxa contributed to procure a temporary recovery. The patient afterwards became ill, and died; and, on dissection, it was found that a large quantity of serous fluid had been effused underneath the mucous membrane of the upper opening of the larynx, so as forcibly to raise or detach the membranes from the epiglottis and arytenoid cartilages. The aryteno-epiglottidean folds were swollen and œdematous; the epiglottis so thickened and rigid, as scarcely to be susceptible of the slightest elevation, or depression; a foul ulcer, the size of a shilling, was situated on the inner surface of the trachea, immediately below the cricoid cartilage, and the larynx and trachea were "filled" with viscid mucus. In cases of laryngeal ulceration, we have frequently procured benefit, a decided mitigation of symptoms at least, from a repeated inspiration of acidulous steam: this is obtained by throwing a suitable proportion of good wine-vinegar, or lime juice, into a deep narrow vessel, filled with boiling water; to this, a few drops of the essential oil of lemons may be added; and the patient's head, as well as the vessel, should be covered with a light close cloth, while the vapour is being inhaled.

On the failure of all other means, Mr. C. proposes the operation of tracheotomy, as a last resource in the laryngeal ulceration, and advises its performance before those symptoms arise which indicate an affection of the lungs. The preferable mode of doing this operation, he says, is by the removal of a portion of the rings of the trachea, either by the knife or scissors; this admits the patient to breathe freely, allows an easy passage for the mucous discharge, and, at the same time, induces that favourable state of quiescence which is necessary to the ulcer's healing.

Pains in the joints, and nodes of the bones, come, in the last place, under our consideration; they are to be treated as in the pustular venereal disease. In the phagedenic form, inflammation and swelling of the knee-joint occur most frequently, and require the most active antiphlogistic measures, such as frequent local depletions, by means of leeches or scarification, with warm fomentations and poultices. When the acute symptoms have been reduced, blisters are serviceable, and the tartarized antimonial ointment. "Mercury, imprudently prescribed, and obstinately continued" determines the worst effects; in numerous instances, Mr. C. has found this symptom yield, in the most satisfactory manner, without the exhibition of a single grain of that mineral; and, generally speaking, he adds, there is no application equal to the tartarized antimonial ointment, for the removal of chronic venereal pains; but, at the same time, the constitutional treatment, comprehending the warm bath, should not be neglected.

Nodes of the bones, our author hopes, will become more and more rare, according as the inveterate practice *(mercury prematurely)* by which they are occasioned, gives place to the modern improvements. He is not certain that they do occur in the phagedenic venereal disease, when mercury has not been employed; but, if they do, leeches and repeated blisters generally relieve the pain, and reduce the swellings. Should, however, notwithstanding these means, the nodes continue obstinate, and the pains severe, recourse must be had to mercury. Periostitis, no matter from what cause it may originate, is powerfully combated by the proper mercurial influences, which check the adhesive inflammation. Mr. C. prefers calomel with opium, so as to affect the gums, but employs inunction, when the digestive organs are delicate; on failure of these remedies, he practises a free division of the inflamed periosteum, over the node. He has never seen enlargement of the testes, in this form of the disease, except when mercury had been previously administered; he treats it with leeches, counter-stimulants, sarsaparilla, and antimony; and, when these fail, with calomel and opium, or cicuta, in alterative doses. As a concluding summary, he adds, the treatment recommended for this, as well as every other form of the venereal disease, is, in fact, founded upon general pathological principles, and the success which has attended these views, is neither *obscure nor incomprehensible;* but that they yield to the treatment which a plain sound pathology points out, and which is available against the other diseases to which they bear an analogy.

After submitting a few very sensible practical remarks, in further illustration of his views, Mr. C. passes on to the detail of fourteen cases, exemplifying the injurious consequences attendant upon the premature exhibition of mercury for the constitutional symptoms of the phagedenic disease; but many of them, also, illustrating the beneficial effects of that medicine, when the disease is on the wane. Thirteen of them furnish evidence of the efficacy of the plan of treatment recommended for the constitutional symptoms of the phagedenic disease, many of them, at the same time, exhibiting the primary symptoms. No. XLIV. is an instance of the usual fatal termination of venereal ulceration of the larynx; XLV. XLVI. and XLVII. explain the plan of treatment preferred when the nasal passages are affected. All of these cases are very valuable; some of them remarkably instructive, and we decline transferring an outline of them to our pages, with the utmost reluctance. This, however, is of the less importance, as we do not conceive that any practitioner, after perusing our accouut of Mr. C's excellent essay, will permit a single month more to elapse, before procuring the work for his own minute and repeated consultation.

Mr. C. closes this, the fifth, chapter of his book, with a reference to the Edinburgh Review (*Med. and Surg. Journ. for July* 1825) of his first edition, and a reply to the objections taken by Mr. Guthrie, to some of his particular views and deductions. The former contains a due proportion of soft and hard epithets; the latter is manly and temperate, but sufficiently distinguished by the author's characteristic manner of philosophizing, and by his enthusiasm. As, however, we are indifferent lovers, even of generous strife, we shall hasten to the more agreeable consideration of our author's fourth modification of the venereal disease.

CHAP. VI.—*Scaly Venereal Disease.* Mr. John Hunter describes the *primary* syphilitic ulcer which, Mr. C. says, gives origin to the scaly eruption, in these words,—"it is somewhat of a circular form, excavated, without granulations, with matter adhering to its surface, and with a thickened edge and base; this hardness, or thickening, is very circumscribed; not diffusing itself gradually and imperceptibly into the surrounding parts, but terminating rather abruptly." This description conveys an exact definition of chancre; so far, at least, says Mr. C. as it occurs on the glans and prepuce, though not on the body of the penis, where a slight difference is observable. Ulcers which are not syphilitic, may, but seldom have a fullness, and slight induration round the circumference; but then, our author adds, this induration has not the firmness of a real chancre, neither does it terminate abruptly, but diffuses itself gradually and imperceptibly into the surrounding parts; in this respect, it differs from chancre so evidently, as to be at once recognized. The induration of a chancre is not confined to the edges only; it extends under the entire surface of the ulcer; frequently, indeed, the ulceration is inconsiderable in comparison with the extent of the induration; and many instances occur, of an indurated knot, or tubercle, on the penis, without any visible ulcer, although these were followed by the constitutional symptoms of syphilis. It is probable, however, that in every such instance, a small ulcer existed, at first, on the callous part, which healed under the use of some local application. Chancre, says the author emphatically, is, in fact, an indolent ulcer, and makes but slow progress, compared to those ulcers of the parts of generation which are destitute of any surrounding induration, and particularly the phagedenic and sloughing ulcers.

Frequent irritation is capable of producing considerable induration around the most simple sore: in forming our diagnosis respecting chancre, we should, therefore, always take into consideration the previous management of the ulcer. When situated on the body of the penis, the chancre has a dark livid colour; the ulcer forming it is level with the surrounding parts, and has less induration than such as are excavated; its edges are a little raised, with the encircling hardness very distinct; and it varies from the size of a sixpence to that of a half-crown, sometimes even extending round the body of the organ. If mercury be administered it soon assumes a healthy appearance; but, if that medicine is withheld, the livid surface of the sore alternates every third or fourth day, with that of a bright brown or tawny colour. At the same time, its dimensions slowly extend, and the encompassing induration obviously increases.

Mr. C. illustrates the preceding description, with a case which is quite apposite. We then find him remarking, that the dark livid appearance of this chancre, when extensive, bears some resemblance to that of the sloughing ulcer, and may possibly be mistaken for it: the indurated edge and base of the chancre, however, do not accompany the latter; and, on a close inspection, the surface of the chancre, though dark, does not exhibit a slough or mortification. Its progress, also, is slow, and a little delay never fails to disclose the ulcer's true nature: if *chancrous*, it will, in a few days, assume a tawny appearance; and, if *sloughing*, the destructive process will extend, or the sloughs separate, and expose to view a corroding or really phagedenic ulcer.

Phymosis and inflammation are less frequently symptoms of chancre, than of the other varieties of ulcer; when, however, they do attend it, the circumstances ought probably to be ascribed to an irritable habit, or some irregularity of life, rather than to any particular quality of the poison by which the disease is induced.

We quote the following paragraph, as it contains a fair attempt at originality.

"It is not very likely," our author observes, at p. 309, "that the distinction existing between syphilitic buboes, and those from other causes, before ulceration takes place, will ever be discovered; and, even in the ulcerated state, it is almost impossible to point out, with any certainty, such discriminating marks as will clearly distinguish them. The following circumstances, in conjunction with the history of the disease, may assist in forming a diagnosis. Syphilitic buboes have frequently aching pains, in which they differ from indolent ulcers of the groin, which seem to maintain themselves by habit. The bottom of the syphilitic bubo, which has not been affected by mercury, has frequently a callous feel, and is either of a dark, foul appearance, or of a light brown tawny colour. If an ulcer of this kind spreads, we may, with confidence, have recourse to mercury, and we shall, in most instances, find that quick amendment follows its exhibition. If the tumour in the groin has not been preceded by an ulcer on the genitals, mercury is unnecessary, and may be highly injurious. I am not in the habit of ordering that medicine for buboes which have not been preceded by chancre; yet the subsequent appearance of syphilitic constitutional symptoms has never, in any instance, occurred; or any other circumstances, to induce me to repent of this line of conduct."

When the syphilitic poison has been absorbed into the system, the *first constitutional* symptoms appear upon the skin, throat, or mouth, and afterwards on the periosteum, bones, and other deep-seated structures. Usually, the cutaneous eruption is considered the first, but ulceration of the throat is as frequently the earliest intimation of the disease having become general. Previously, however, to these local signs, the patient's countenance grows dull and pallid, and he complains of restlessness, want of sleep, head-ache, and other feelings which announce derangement of the constitution. This derangement, in Mr. C's mind, is analogous to the fever which precedes the eruption in the other kinds of venereal, and the exanthematous diseases. In the latter, the fever diminishes as the eruption advances, and the constitution supports itself by its own powers; but, in syphilis, it is slow, and has much of the hectic nature; it may subside, on the first symptoms appearing; but, the irritation from the unsubdued poison still remaining, the same effort for relief is again and again renewed; in other words, says our author, the fever returns, but at uncertain intervals, till the constitution is worn out, or the intervention of medicine excites an action sufficient to supersede the syphilitic irritation.

An efflorescence of the entire skin, giving it a mottled red appearance, frequently precedes the syphilitic eruption; it is *scaly*, and therefore quite distinct from those connected with the other forms of the disease; Mr. C. has never observed it to be *papular*, *pustular*, or *tubercular*, when it arose from the true syphilitic primary ulcer; nor to be *scaly*, when it followed those ulcers which do not possess the characters of chancre, the indurated edge and base: a few pimples on the face of a person having the *scaly* eruption, ought not to be reckoned an exception; the appearance of the entire surface of the body should be taken into consideration. It is found, in almost every instance, on the forehead, breast, back of the neck, the groins, and surface adjoining the pubes: such spots as are situated near parts covered with hair, spread into each other, and give rise to large copper-coloured blotches. On the palms of the hands, or soles of the feet, the cuticle separates, and is quickly regenerated; and this process may be several times repeated, for the thick skin of these parts has not a disposition to form scurf. On this account, if a case should occur, in which the appearances were confined to the hands and feet, it would be impossible to determine, whether or not it were syphilitic. When the eruption affects a skin opposed by another skin, as between the nates or the scrotum and thigh, as under the arm, or between the thighs, it is not scaly; but the skin rises into a moist, soft, flat, or convex surface, which discharges a whitish matter. In this stage of the disease, that part of the fingers or toes on which the nail is placed, becomes affected, and a separation of the nail follows; but, of course, there cannot be a succession of this, as of the cuticle. When mercury has not been employed, the eruption proceeds to ulceration, in the following manner:—each spot is covered by scales or scurf, which is thrown off, and succeeded by another; and every new scurf becomes thicker than the preceding, till, at length, it forms a crust, under which matter collects, and gives origin to a true ulcer, that slowly spreads.

Ulceration of the throat, is the *next* constitutional symptom of the scaly venereal malady. In most instances, the tonsil is the seat of the ulcer, which forms without much previous pain and swelling, although it soon produces a considerable excavation. As the tonsils, however, are sometimes deeply ulcerated in the phagedenic disease, the true nature of the ulcer, and of the case, shall be best ascertained by a careful examination of the patient, and the history of his morbid state. Chronic inflammation of these glands, with irregularity of their surface, which is covered with coagulable lymph, may give the appearance, but it is the appearance only, of ulceration of the parts. Another source of deception would arise from an inflamed and ulcerated condition of the fauces, occasioned by exposure to cold and wet, during the use of mercury, did not the peculiar fetor of the breath discover the character of the affection. Ptyalism not unfrequently supervenes, in patients who have not taken mercury; but, in general, these persons have venereal ulcers of the fauces, at the same time. Mr. C. thinks it probable, that this spontaneous salivation is a constitutional symptom of syphilis, and analogous to a similar occurrence in small-pox. He is not inclined to attribute it to the irritation of the ulcers of the throat, upon the mouth, and salivary glands; for, he says, the syphilitic ulcers in question, are attended with scarcely any pain. Now, it is possible that this conclusion may be the right one; but, to our mind, it exhibits a genuine instance of the manner in which the process of philosophizing may be influenced by the enthusiasm of particular views:—when visible effects are attended by operating causes capable of producing them, and these causes themselves are also manifest, we would prefer retracing such effects to the palpable, rather than to a conjectural, origin. Mr. C. reckons it a curious circumstance, but one which he would naturally expect from his view of their nature, that as the patient, in these cases, becomes affected with mercury, the salivation begins to diminish, and, at length, entirely ceases; but is renewed again, as the mercurial process is farther advanced; first, arising from the disease, and afterwards from the remedy:—we take the explanation of this from what is observed in daily experience,—the action of an artificial disease, superseding that of the natural.

The bones, periosteum, fasciæ, and ligaments, are the deep-seated parts most liable to attacks of the syphilitic poison; and it is generally thought, our author remarks, that they are affected after the disease has appeared constitutionally on the superficial parts of the body, if it has not been arrested in its progress by mercury, which disturbs the regular succession of the symptoms: we are unacquainted, however, with the evidences of this distinction. Bones nearest the surface, as the tibia, sternum, clavicle, and cranium, are most liable to this disease, the advances of which are so much the more gradual, inasmuch as the affected parts are deep seated. In most cases, the true syphilitic node is a very indolent swelling, increasing by slow degrees, and being attended with as little pain and inflammation, as discolouration of the integuments, until, in an advanced state. *Syphilitic* pains are generally supposed to affect the centre of the long bones, while those of the *resembling* diseases occupy the joints; but, our author justly observes, this is not universally the case; in common with all other aches or pains, they have their exacerbation at night.

Mercury, Mr. C. informs us, subdues the symptoms of syphilis, with greater rapidity and certainty than takes place in chancre. The eruption begins to decline, even before the mercurial impregnation of the system is observed; and, as soon as this is established, the ulcers of the surface and throat begin to granulate, and forthwith cicatrize, and elevations of the skin between the nates, under the scrotum, and in the arm-pits, as well as the ulcers of the tonsils, all immediately amend. Syphilitic nodes, however, do not yield to this medicine with the same regularity and quickness, as the constitutional affections of the surface: Mr. C. thinks this may probably be owing to the low organization of the bones. He doubts the propriety of continuing a full mercurial action longer than two months, which period he reckons more than sufficient to supersede the syphilitic action, if managed with judgment. We submit the following extract to the reader's consideration.

"Syphilis," Mr. C. observes, p. 321, "like other contagious diseases, is obedient to certain laws, from which there is little deviation; but, from the details of most authors, it would appear that it possesses but little regularity in its progress. This opinion of the multiform appearance, and irregular course of the disease, is occasioned by several circumstances: 1st. Ulcers on the genitals, which are not syphilitic, are frequently mistaken for syphilitic, and treated accordingly; and this is a source of error, not only in respect of the original disorder, but in the entire train of complaints which may arise from the distemper thus mistaken. 2d. If the primary complaint is syphilitic, the progress of the disorder is interrupted by the use of mercury, and if it is not totally subdued by that remedy, its return occurs at uncertain intervals. 3d, There are a number of complaints, which arise from the use and abuse of mercury, which are frequently mistaken for the symptoms of syphilis; for instance, erratic pains, diseases of the viscera, nervous affections, mania, fatuity, and a variety of other complaints, which can evidently be traced to the imprudent use of this medicine. In the treatment of true syphilis, there is nothing perplexing or dubious, if we except the mercurial phagedena and other effects of that mineral, which may be mistaken by the inexperienced, for the symptoms of syphilis: but the remedy, in the hands of the experienced practitioner, will remove, I may say, with certainty, every symptom, both local and constitutional."

For superseding the syphilitic action, Mr. C. is of opinion, that the maintaining a strong mercurial irritation in the system for one or two months, is all that is required: alterative courses may suspend, but they will seldom expel the poison. He cautions his readers against exhibiting one additional grain beyond what is necessary for producing this effect; and also, very properly advises taking the warmth and mercurial atmosphere of an hospital into consideration, as calculated to determine modifications in the result different, at least in regard to time, from what shall be obtained in private practice. With some excellent observations on the bad consequences of mercury, real or conjectural, and three valuable cases, Mr. C. brings us to a recapitulation of the drug's usual and characteristic effects on the frame, and likewise those effects which now and then proceed from its use, and which seem to originate in peculiarity of constitution.

"1st. Mercury," says he, p. 334, "induces a specific fever, different from all others, and attended with an increase of the various secretions. 2d. When the constitution has been incessantly harrassed by mercury, it (the mercury) induces dropsy, various nervous affections, epilepsy, mania, and fatuity. 3d. It produces peculiar local effects. A crude wound, or suppurating sore, under its influence, will immediately become a spreading phagedenic ulcer, having a fiery red appearance. The ulcers of morbid poisons, after the peculiar action of their respective poisons has ceased to act, may become, in the same manner, mercurial ulcers; but, if the poison retains its influence in any portion of the ulcer, as soon as the mercurial phagedena has subsided, it may infect the remainder of the ulcer, which will consequently re-assume its original character. 4th. It occasionally produces pains resembling rheumatism, and swellings of the joints, particularly when the patient exposes himself to cold. 5th. It is asserted to be capable of producing nodes, which resemble the syphilitic; but this I doubt, because there is no authenticated instance of nodes of the bones occurring under courses of mercury, for any diseases except venereal. 6th. It produces two affections of the constitution; the mercurial eruption and mercurial erethismus, which unlike its usual and characteristic effects, are evidently owing to some peculiarity of constitution. In the same manner, most medicines, and many of our common aliments, produce phenomena in some constitutions attended with great disorder of the system, totally different from their accustomed and well-known effects. 7th. Mercury, and more particularly a mercurial atmosphere, are in the highest degree prejudicial and dangerous to patients labouring under any pulmonary affection, by producing a rapid state of excitement, and consequent effusion in the lungs and chest."

Mr. C. concludes this branch of his subject, with a few remarks and illustrations of the question, whether every form of venereal disease, including syphilis itself, which he calls the *scaly* form, can be successfully treated without mercury. His experience, he says, has afforded sufficient proofs, though few, that *all* the varieties of the venereal and syphilitic maladies *can* be cured without the aid of that powerful medicine: but it does not, by any means follow, he adds, that, therefore, the non-mercurial is the most judicious mode of treatment; for, in cases thus treated, the recovery is remarkably slow, both from the primary and constitutional affections. On the contrary, however, the cure is certain and rapid, where mercury is exhibited, according to our author's rules, for the true syphilitic symptoms.

Chap. VII.—This relates to *diseases most likely to be confounded with those of venereal origin*, and at the same time comprehends some good practical observations on the phlegmonous and erysipelatous inflammations of the penis; on herpes præputialis; ulcers on, or in the vicinity of, the genitals; and certain cutaneous affections. We cannot resist the inclination to transcribe Mr. C's. sentiments regarding a disease, which is as prevalent as its distinctive symptoms are indefinable.

"On another occasion," *(in his Essay on Scrofula)* says he, p. 351, "I have adduced incontrovertible facts which demonstrate, that disorder of the chylopoietic viscera precedes and accompanies the symptoms of scrofula, and that there are the strongest grounds for believing, that such disorder is, in a very great majority of cases, the immediate cause of the disease. A defective digestion, continued for any length of time, must as certainly produce chyle, or blood of a vitiated quality, and unfit to replenish the waste of the body, as the constant use of unwholesome food, which is undergoing the putrefaction and acetous fermentation. A disordered state of the system at first ensues, and is followed by various local complaints. Among these, are swellings of the lymphatic glands, which frequently ulcerate, different kinds of cutaneous affections, particularly the tubercular eruption, which likewise ulcerates. Ulcers are often seen in the throat, which it is not easy to distinguish from those of venereal origin; and, lastly, ulceration of the pituitary membrane is very frequent, attended with caries of the bones of the nose and palate, and often with fistula lachrymalis. Even the deep-seated parts do not escape; for the patient complains of pains in the joints, which are often mistaken for those of chronic rheumatism; and affections of the bones or their coverings, producing a kind of node, are not unfrequent. In fact, this train of symptoms occur every day under our eyes in children, whose youth fortunately protects them from the suspicion that lights on their elders; for it is by no means unusual to find, in scrofulous children, ulcers which arise from tubercles, and also ulcers of the tonsils, nodes on the shins, and pains in the joints, which, if they occurred at a more advanced period of life, would inevitably subject them, in the hands of a great majority of practitioners, to a severe course of mercury."

Having finished his concluding remarks, our author makes this statement,—that, after several years of observation and scrutiny, during which these diseases were constantly and carefully watched and followed, he has not met with any circumstance to induce him to doubt the principles he endeavours to enforce in his writings; and observes, in fine, that a classification of venereal complaints, grounded on the character of the eruption, is not only the most natural and most in accordance to the pathological arrangement of other eruptive diseases attended with fever, but it is also, in a practical point of view, the most useful that can be devised; for the general tendency of the disease, with respect to mildness, severity, or duration, may be anticipated by the character of the eruption.

Mr. C. finishes this remarkable book, with a synoptical recapitulation of the practical doctrines, which it is the chief object of the volume to unfold: the advantages of such a scheme are manifest, and we feel desirous of putting our readers in possession of them; but this, our limits altogether forbid. Here, then, we bring this long, though we trust not unuseful, article to a conclusion.—Our respect for those who promote the diffusion of medical knowledge, through the instrumentality of the press; our regard for the indefatigable author of the Essay we have been considering; and our own zeal for the best interests of the most beneficent of all the sciences, have prompted us to go into an elaborate explication of Mr. Carmichael's peculiar views and practices; and, in executing this important task, we have proved ourselves the instruments, we hope, of communicating much instructive experience, and of offering, at the same time, an invitation to practitioners, in almost every region of the civilized world, to subject these inductions and doctrines to diversified probation, so as to modify or confirm their accuracy; and, by consequence, to determine the degree of their usefulness. On the present occasion, in fine, we have made ourselves the historians rather than the judges of Mr. C's philosophy; but we have, nevertheless, examined it with candour and impartiality: while, therefore, we freely avow sentiments of grateful courtesy towards its author, who, except by his writings, is perfectly unknown to us, we would very earnestly beseech every member of the profession, to regard him and his extraordinary Essay with the same spirit; to entertain a kindliness of feeling towards the labours of such a man, which, though they may be utterly repugnant to pre-established opinions, cannot possibly have other than charity and disinterestedness for their object; and, indeed, to encourage him, and all of us, to persevere in the course he has entered, as a generous champion of truth and philanthropy.

---

